# Mechanochemical Aza-Vinylogous Povarov Reactions for the Synthesis of Highly Functionalized 1,2,3,4-Tetrahydroquinolines and 1,2,3,4-Tetrahydro-1,5-Naphthyridines

**DOI:** 10.3390/molecules26051330

**Published:** 2021-03-02

**Authors:** José Clerigué, M. Teresa Ramos, J. Carlos Menéndez

**Affiliations:** Unidad de Química Orgánica y Farmacéutica, Departamento de Química en Ciencias Farmacéuticas, Facultad de Farmacia, Universidad Complutense, 28040 Madrid, Spain; joscleri@ucm.es (J.C.); mtramos@ucm.es (M.T.R.)

**Keywords:** nitrogen heterocycles, vibratory ball milling, tetrahydroquinolines, tetrahydro-1,5-naphthyridines

## Abstract

The aza-vinylogous Povarov reaction between aromatic amines, α-ketoaldehydes or α-formylesters and α,β-unsaturated dimethylhydrazones was carried out in a sequential three-component fashion under mechanochemical conditions. Following extensive optimization work, the reaction was performed on a vibratory ball mill operating at 20 Hz and using zirconium oxide balls and milling jar, and afforded 1,2,3,4-tetrahydroquinolines and 1,2,3,4-tetrahydro- 1,5-naphthyridines functionalized at C-2, C-4 and also at C-6, in the latter case. This protocol generally afforded the target compounds in good to excellent yields and diastereoselectivities. A comparison of representative examples with the results obtained under conventional conditions revealed that the mechanochemical protocol affords faster Povarov reactions in comparable yields using a solvent-less environment.

## 1. Introduction

**Figure 1:**
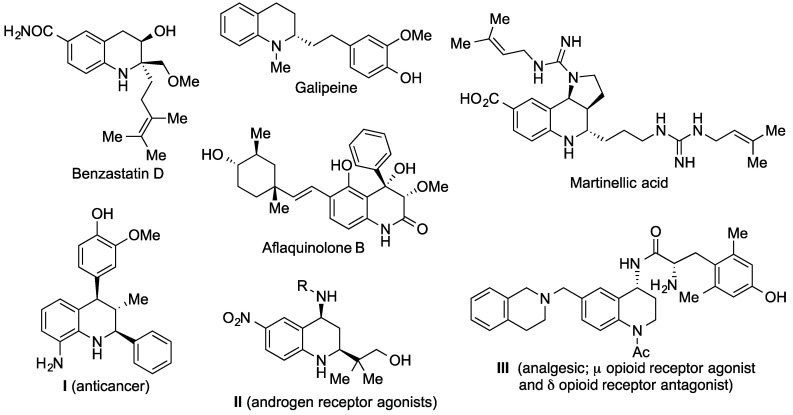
Selected natural and synthetic 1,2,3,4-tetrahydroquinolines.

1,2,3,4-Tetrahydroquinoline is one of the most relevant simple heterocyclic systems, being the structural core of many natural products such as the benzastatins [1], galipeine [2,3], the aflaquinolones [4] and martinellic acid [5], among many others (Figure 1). Moreover, a large number of synthetic tetrahydroquinolines with interesting pharmacological properties are also known [6,7], including the representative examples **I**–**III** shown in Figure 1.

**Scheme 1:**
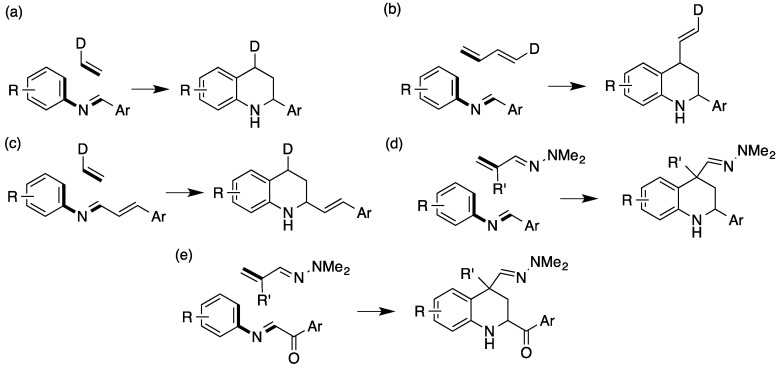
Some variations of the Povarov reaction: (**a**) The standard Povarov reaction. (**b**) Type I vinylogous Povarov reaction. (**c**) Type II vinylogous Povarov reaction. (**d**) Type I aza-vinylogous Povarov reaction. (**e**) Synthesis of 2-acyl-1,2,3,4-tetrahydroquinolines by application of the Type I aza-vinylogous Povarov reaction.

Because of their significance in drug discovery, the development of new methodologies for the synthesis of tetrahydroquinoline derivatives is a very active area [6,7]. Nevertheless, access to highly functionalized derivatives of this scaffold is still challenging owing to poor functional group compatibility of many of the known synthetic methods. One of the best-studied routes to tetrahydroquinolines is known as the Povarov reaction, and can be defined as a formal [4 + 2] inverse electron demand cycloaddition between aromatic imines and electron-rich olefins (Scheme 1a) [8,9]. A variety of post-condensation transformations may be performed on Povarov adducts, depending on the type of substituents and functional groups present in the structure. In this connection, vinylogous Povarov reactions, i.e., those where an extended unsaturated moiety is present in the dienophile (Type I reactions) or in the imine (Type II reactions) [10], are particularly interesting because they allow to obtain tetrahydroquinolines with an olefin substituent at C-4 or C-2, respectively (Scheme 1b,c). A more direct, although relatively unexplored, approach to functionalized tetrahydroquinolines involves the use of dienes or dienophiles bearing functional groups. Thus, we have developed a Povarov reaction that furnishes tetrahydroquinolines bearing a quaternary stereocenter at C-4 attached to a dimethylhydrazone group and aryl substituents at C-2 by employing an α,β-unsaturated hydrazone as the dienophile (Scheme 1d) [11,12]. Later, we discovered that this reaction can be performed starting from α-ketoimines, giving ready access to 2-acyl-4-dimethylhydrazono-1,2,3,4-tetrahydroquinolines (Scheme 1e) [13]. Regarding 1,2,3,4-tetrahydro-1,5-naphthyridines, although less developed than tetrahydroquinolines, they are very attractive due to their potential drug-likeness, which has prompted some synthetic efforts towards their preparation [14].

Sustainable chemistry, i.e., the design of chemical processes to minimize the handling and generation of hazardous compounds, has become in recent years one of the main criteria by which the efficiency of synthetic routes is evaluated. In pursuit of this goal, previously known transformations often need to be redesigned in order to minimize the environmental consequences of their use. Solvents have been traditionally considered essential for achieving homogeneous energy transfer in organic reactions, besides sometimes having a role in the rate and course of reactions. As pointed out by Tanaka and Toda in their seminal 2000 review [15], most known organic reactions have been developed in solution because historically it was believed that matter can only be transformed in solution state, a concept that can be traced back to the Greek philosopher Aristotle (*corpora non agunt nisi fluida seu soluta*, i.e., compounds do not react unless dissolved) but has proved incorrect. Although their use is standard in synthesis, solvents pose a number of safety problems such as toxicity and fire risks, and they are the main source of residues from synthetic operations, both in academic and industrial settings, since their mass can constitute up to 90% of reaction mixtures [16]. For these reasons, and also because of their high cost, there is much current interest in replacing conventional solvents by green alternatives [17,18] and even more so in the development of solvent-free methodologies [19,20,21]. Indeed, solvent-free reactions satisfy at least two of the 12 green chemistry principles, namely “safer solvents and auxiliaries” and “waste prevention” [22]. Synthetic transformations in the absence of a solvent can be achieved by using one liquid reactant as solvent (neat reactions), but this strategy poses some problems due to the need to achieve a homogeneous mixing in spite of the high viscosity of the reaction medium. Alternatively, solvent-free reactions can be performed in the solid state, which, besides the above-mentioned environmental aspects, may provide additional advantages due to the very high reagent concentrations prevalent under solid state conditions such as higher reaction rates and the possibility to uncover new modes of reactivity. Mechanochemical activation is the main current approach to solid-state synthetic chemistry and relies on the application of mechanical energy, produced from grinding or milling processes, to achieve chemical transformations [23,24,25,26,27,28,29,30,31].

In spite of the importance of the Povarov reaction, its mechanochemical variation has received very little attention. To our knowledge there have been only two reports on this area, with a limited number of examples and no attention to the vinylogous case. Thus, Zang et al. described a FeCl_3_-catalyzed mechanochemical version of the Povarov reaction that yielded *cis*-2,4-diphenyl-1,2,3,4-tetrahydroquinolines when styrenes were employed as the dienophile component [32] or fully aromatic quinolines when starting from phenylacetylenes [33]. Similarly, Kouznetsov and co-workers reported the application of vibratory milling to the Povarov reaction starting from vinyl acetamides and using phosphomolybdic acid as catalyst [34].

In this context, we describe here the development of mechanochemical conditions for the aza-vinylogous reaction between α,β-unsaturated hydrazones and α-ketoimines.

## 2. Results and Discussion

**Scheme 2:**
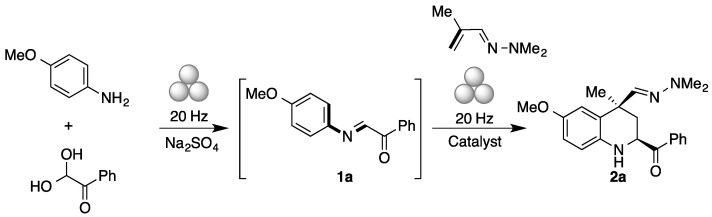
Model reaction studied during the optimization work.

We started our study by establishing the conditions for the mechanochemical generation of the intermediate imine, using as a model the reaction between *p*-methoxyaniline and phenylglyoxal. Because the latter compound is available commercially as a hydrate, and also bearing in mind the liberation of water from the reaction, we performed the reaction by milling together the starting materials and anhydrous sodium sulfate, to serve as both a dehydrating agent and a milling assistant. Under high-speed vibration milling (HSVM) at 20 Hz [35], using a zirconium oxide milling jar and a single 20 mm ball of the same material, the *gem*-diol dehydration/imine formation process required 75 min to achieve complete conversion. The formation of imine **1a** was verified by NMR in the initial experiments, but for our routine optimization work the in situ preparation of **1a** was followed by simple addition of methacrolein dimethylhydrazone and a catalyst to the reaction vessel followed by additional milling at 20 Hz, thus leading to the synthesis of **2a** by a one-pot sequential multicomponent protocol (Scheme 2, Table 1). An initial experiment in the absence of any additive (entry 1) gave a poor yield, confirming the need for a catalyst. We studied the effect of a 10 mol% concentration of a number of Lewis acids, including Ce(IV) ammonium nitrate, CAN (entry 2), iron trichloride (entry 3), aluminum trichloride (entry 4), scandium triflate (entry 5), ytterbium triflate (entry 6), Eu(hfc)_3_ (entry 7), boron trifluoride etherate (entry 8), zinc chloride (entry 9) and indium trichloride (entry 10). Since the latter catalyst gave the best results (90% yield and ca. 7:3 dr), some additional experiments were performed, including the use of 20% catalyst (entry 11), the use of a 16 mm ball (entry 12) and a higher vibration frequency of 25 Hz (entry 13), without observing improvements. Some attempts were also made to replace zirconium oxide by stainless steel (entries 14–16) and vibratory milling by planetary ball milling (PBM) (entries 17 and 18), again without any enhancement in yield or dr.

In order to complete the optimization study, we decided to examine the effect of Brønsted acids. While camphorsulfonic acid did not provide any improvement (entry 19), *p*-toluenesulfonic acid gave the same yield as indium trichloride (90%) and a slightly improved 75:25 diastereomeric ratio (entry 20). Two subsequent experiments confirmed 60 min to be the optimal reaction time (entries 21 and 22), and an increase of the catalyst load to equimolecular levels was not beneficial (entry 23). Two additional experiments showed that the reaction can be scaled up to 3 mmol with a lower but still acceptable yield (entry 24), and finally, that work-up was necessary to maintain good yields (entry 25). Due to the lower cost and higher stability of *p*-toluenesulfonic acid in comparison with indium trichloride, we chose it as a catalyst in our studies of the scope of the method. Finally, we also performed this reaction under mortar and pestle conditions for 1 h, finding that compound **2a** was formed to some extent, but the product was much more impure than in the corresponding ball milling experiment.

**Scheme 3:**
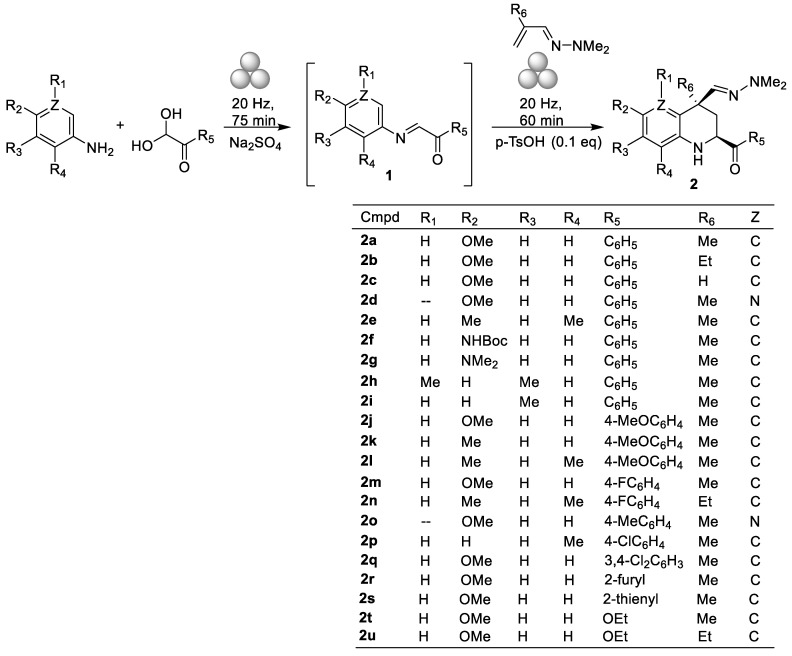
Sequential multicomponent synthesis of 1,2,3,4-tetrahydroquinolines and 1,2,3,4-tetrahydro-1,5-naphthyridines under ball milling.

Using the optimal conditions described in entry 20, we set out to examine the generality of the mechanochemical protocol (Scheme 3). The reaction required an electron-releasing group (Me, OMe, NHBoc, NMe_2_) at the aniline aromatic ring to proceed, which would end up placed at the C-6, C-7 or C-8 positions of the tetrahydroquinoline ring, but the presence of a C-5 substituent led to a poor diastereoselection, as observed in the case of **2h** (see the discussion corresponding to Table from the Scheme below). On the other hand, the reaction was compatible with both electron-withdrawing and electron-releasing substituents at the C-2 benzoyl, and also with the presence of heterocyclic substituents (compounds **2r** and **2s**) and an ester group (compounds **2t** and **2u**). Finally, the R^6^ substituent at C-4 could be hydrogen or a small alkyl group. Interestingly, the reaction also worked well for substrates containing a lactim ether moiety at the aromatic ring of the starting aromatic amine, affording 1,2,3,4-tetrahydro-1,5-naphthyridines **2d** and **2o** in full regioselectivity. The 1,5-diaza structure was confirmed by the value of the coupling constant of 8.6 Hz, corresponding to the ortho coupling between H-7 and H-8.

As shown in Table 2, the mechanochemical conditions gave generally good to excellent yields. They were comparable to the ones observed in solution, with improvements being observed in some cases (e.g., **2a**, **2h**, **2m**). Diastereoselection was generally in the 2.5:1 to 6:1 range, in favor of the isomer having a *cis* relationship between the two functional groups and was slightly worse than the one obtained in solution. The only exception was compound **2h**, which was obtained with no significant diastereoselection under both methods. This was ascribed to steric compression between the C-4 and C-5 methyl substituents when the former is equatorial, leading to a similar stability for the *cis* and *trans* isomers. Importantly, the mechanochemical method had the advantage of much shorter reaction times in comparison to the solution conditions. The slight drop in diastereoselectivity under mechanochemical conditions may be due to an increased reaction temperature due to the milling process.

The *cis* relative configuration of the major diastereomer was established in our earlier solution chemistry work on the basis of NOE and single crystal X-Ray diffraction experiments [12].

**Scheme 4:**
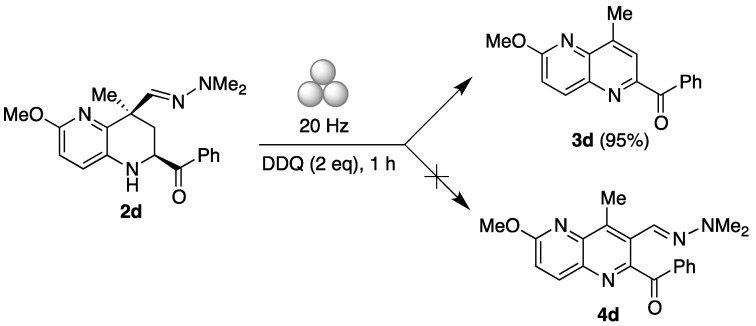
Oxidative aromatization of 1,2,3,4-tetrahydro-1,5-naphthyridine **2d** with loss of the dimethylhydrazonomethyl substituent.

Finally, we briefly examined the potential application of the 2-acyl-1,2,3,4-tetrahydro-1,5-naphthyridine derivatives to the synthesis of 2-acyl-1,5-naphthyridines, a class of compounds that are of potential pharmaceutical interest but whose preparation requires long sequences [36]. We have previously described several methods for the transformation of aza-vinylogous Povarov products into 2-acylquinolines, and were interested in studying the application of this chemistry to the 1,5-naphthyridine case, comparing conventional solution chemistry with mechanochemical conditions. To this end, we examined the reaction of compound **2d** with magnesium monoperoxyphthalate, which, according to our precedent, should lead to aromatization with concomitant loss of the dimethylhydrazono group via a sequence of reactions comprising N-oxidation/nitrile formation via a Cope-type reaction/hydrogen cyanide elimination/dehydrogenation steps [13]. However, either 6 h reflux in solution or 1 h under ball milling led only to ca. 50% conversion of **2d** into the corresponding 2-acyl-1,5-naphthyridine **3d**. On the other hand, treatment of **2d** with DDQ afforded a quantitative yield of **3d** under vibratory ball milling (20 Hz, ZrO_2_ ball and jar), and a complex mixture containing only a small amount of **3d** when the reaction was performed in methanol solution. It is interesting to note that the formation of compound **4d** might have been expected in this experiment, since a C_4_-C_3_ rearrangement of the dimethylhydrazono group followed by dehydrogenation was observed when tetrahydroquinolines were employed as starting materials [37], although these reactions seemed to require a high electron density in the benzene ring of the starting material. Thus, the combination of two mechanochemical steps can be used to synthesize a 2-acyl-1,5-naphthyridine in a high yield from very simple starting materials (Scheme 4).

## 3. Materials and Methods

### 3.1. General Experimental Information

All reagents and solvents were of commercial quality and were used as received. Reactions were monitored by TLC analysis, on silica gel-G aluminium plates with fluorescent indicator from Merck (Tres Cantos, Madrid, Spain). Melting points were measured in open capillary tubes using an instrument from Stuart Scientific (Barcelona, Spain) and are uncorrected. Mechanochemical reactions were carried out in an Anton Paar BM500 ball mill (Madrid, Spain) at a frequency of 20 Hz using a 25 mL zirconia grinding jar and a single ball (20 mm diameter, 25.5 g mass) of the same material. The ^1^H-NMR, ^13^C-NMR and CH-correlation spectra were recorded on Bruker Avance instruments operating at 250 or 300 MHz for ^1^H-NMR (Bruker, Rivas-Vaciamadrid, Madrid, Spain) and maintained by the NMR Unit at Universidad Complutense, using CDCl_3_ as solvent and residual non-deuterated solvent as internal standard. Topspin (Bruker, Rivas-Vaciamadrid, Madrid, Spain) or Mestrenova (Mestrelab, Santiago de Compostela, Spain) software packages were used throughout for data processing; chemical shifts are given in parts per million (δ-scale) and coupling constants are given in Hertz (see Supplementary Materials for the spectra of all compounds). IR spectra were recorded on a Cary630 FTIR instrument from Agilent (Las Rozas, Madrid, Spain) with a diamond accessory for reflectance measurements of solid and liquid sample. Combustion microanalyses were performed by the Elemental Microanalysis Unit, Universidad Complutense, on a Leco 932 CHNS analyzer.

### 3.2. General Procedure for the Synthesis of 2-acyl-1,2,3,4-tetrahydroquinolines and 2-acyl-1,2,3,4-tetrahydro-1,5-naphthyridines (Compounds ***2***)

The suitable aniline (1 eq, 0.5 mmol), the suitable glyoxal derivative (1–1.5 eq, 0.5–0.75 mmol) and anhydrous sodium sulphate (5 g) were added to a 25 mL zirconia milling jar with a single zirconia ball 20 mm in diameter. The vessel was fixed to the horizontal arm of a mixer mill and it was shaken for 75 min at a frequency of 20 Hz. Then, a small sample of the reaction mixture was collected to verify the formation of the corresponding imine **1** by ^1^H-NMR (see the Supporting Information). The suitable hydrazone (1.5 eq, 0.75 mmol) and *p*-TsOH (0.1 eq, 0.05 mmol) were added directly to the mill vessel. The mixture was shaken at 20 Hz for 1 h; caution should be exerted to avoid exceeding this time substantially, since some product decomposition may take place. The jar was washed with 2 × 5 mL of methanol, and the resulting suspension was stirred for 10 min to recover all the material from the ground sodium sulphate. The methanolic suspension was filtered through a pad of celite to remove the sodium sulphate, and the solvent was eliminated under reduced pressure. The oily residue was redissolved in ethyl acetate (15 mL), washed with water (15 mL) and brine (15 mL), and the aqueous phase was extracted with ethyl acetate (2 × 10 mL). The combined organic phases were dried over anhydrous sodium sulphate and evaporated. The resulting crude was purified by silica gel flash chromatography, using the mixture of solvents specified in the description of each compound. Using automated flash chromatography, two separations are needed, where the purpose of the second is to produce the pure *cis* isomer. On the other hand, slow manual chromatography allows the separation of the major diastereomer in a single chromatographic operation.

#### 3.2.1. (±)-(2. R,4R)-2-Benzoyl-4-((2,2-dimethylhydrazono)methyl)-6-methoxy-4- methyl-1,2,3,4-tetrahydroquinoline (**2a**)

Prepared from the in situ-generated imine **1a** (0.5 mmol) and (E)-1,1-dimethyl-2-(2-methylallylidene)hydrazine (84 mg, 0.75 mmol). Chromatography: petroleum ether: ethyl acetate 85:15. Yield: 158 mg (90%), as a yellow solid. Characterization data were identical to those described in the literature [13].

#### 3.2.2. (±)-(2. R,4R)-2-Benzoyl-4-((2,2-dimethylhydrazono)methyl)-4-ethyl-6-meth- oxy-1,2,3,4-tetrahydroquinoline (**2b**)

Prepared from the in situ-generated imine **1a** (0.5 mmol) and (E)-1,1-dimethyl-2-(2-methylenebutylidene)hydrazine (95 mg, 0.75 mmol). Chromatography: petroleum ether: ethyl acetate 9:1. Yield: 108 mg (62%), as an orange oil. Characterization data were identical to those described in the literature [13].

#### 3.2.3. (±)-(2. R,4R)-2-Benzoyl-4-((2,2-dimethylhydrazono)methyl)-6-methoxy-1,2,3,4- tetrahydroquinoline (**2c**)

Prepared from the in situ-generated imine **1a** (0.5 mmol) and (E)-2-allylidene-1,1-dimethylhydrazine (74 mg, 0.75 mmol). Chromatography: petroleum ether: ethyl acetate 9:1. Yield: 114 mg (67%), as a red solid. Characterization data were identical to those described in the literature [13].

#### 3.2.4. (±)-(2. R,4R)-2-Benzoyl-4-((2,2-dimethylhydrazono)methyl)-6-methoxy-4- methyl-1,2,3,4-tetrahydro-1,5-naphthyridine (**2d**)

Prepared from the in situ-generated imine **1b** and (E)-1,1-dimethyl-2-(2-methylallylidene)hydrazine (84 mg, 0.75 mmol). Chromatography: petroleum ether: ethyl acetate 9:1. Yield: 173 g (98%), as a pale yellow solid. ^1^H-NMR (300 MHz, CDCl_3_) δ: 8.02–7.96 (m, 2H), 7.67–7.59 (m, 1H), 7.57–7.47 (m, 2H), 7.17 (s, 1H), 7.06 (d, *J* = 8.6 Hz, 1H), 6.53 (d, *J* = 8.6 Hz, 1H), 5.07 (dd, *J* = 12.1, 2.8 Hz, 1H), 4.46 (bs, 1H), 3.86 (s, 3H), 2.69 (s, 6H), 2.29 (dd, *J* = 13.5, 2.9 Hz, 1H), 2.13–1.97 (m, 1H), 1.68 (s, 3H) ppm. ^13^C-NMR (75 MHz, CDCl_3_) δ: 199.9, 157.2, 144.5, 143.4, 135.0, 133.9, 132.8, 129.2, 128.8, 128.6, 109.4, 54.7, 53.5, 43.6, 42.1, 37.9, 27.2 ppm. IR (neat) ν: 3318.5, 2964.1, 1678.3, 1595.4 cm^−1^. Elemental analysis (%): Calc. for C_20_H_24_N_4_O_2_ (M = 352.44): C, 68.16; H, 6.86; N, 15.90. Found: C, 68.50; H, 6.46; N, 15.58. mp: 103–106 °C.

#### 3.2.5. (±)-(2. R,4R)-2-Benzoyl-4-((2,2-dimethylhydrazono)methyl)-4,6,8-trimethyl- 1,2,3,4-tetrahydroquinoline (**2e**)

Prepared from the in situ-generated imine **1c** (0.5 mmol) and (E)-1,1-dimethyl-2-(2-methylallylidene)hydrazine (84 mg, 0.75 mmol). Chromatography: petroleum ether: ethyl acetate 96:4. Yield: 133 mg (76%), as a white solid. Characterization data were identical to those described in the literature [13].

#### 3.2.6. (±)-(2. R,4R)-tert-Butyl 2-benzoyl-4-((2,2-dimethylhydrazono)methyl)- 4-methyl-1,2,3,4-tetrahydroquinolin-6-yl)carbamate (**2f**)

Prepared from the in situ-generated imine **1d** (0.5 mmol) and (E)-1,1-dimethyl-2-(2- methylallylidene)hydrazine (84 mg, 0.75 mmol). Chromatography: petroleum ether: ethyl acetate 7:3. Yield: 157 mg (72%), as a yellow solid. ^1^H-NMR (250 MHz, CDCl_3_) δ: 8.01–7.92 (m, 2H), 7.68–7.59 (m, 1H), 7.57–7.49 (m, 2H), 7.21 (bs, 1H), 6.88 (d, *J* = 2.2 Hz, 1H), 6.73 (d, *J* = 8.5 Hz, 1H), 6.27 (s, 1H), 5.11 (dd, *J* = 12.3, 2.8 Hz, 1H), 4.75 (bs, 1H), 2.78 (s, 6H), 2.11 (dd, *J* = 12.9, 2.8 Hz, 1H), 1.76 (t, *J* = 12.6 Hz, 1H), 1.65 (s, 3H), 1.52 (s, 9H) ppm. ^13^C-NMR (63 MHz, CDCl_3_) δ: 199.8, 153.8, 139.2, 135.1, 134.0, 129.3, 129.3, 129.0, 128.7, 127.4, 120.7, 120.1, 116.6, 80.3, 54.5, 43.7, 41.3, 39.0, 28.8, 26.63 ppm. IR (neat) ν: 3357.8, 2968.6, 1706.2, 1684.5, 1595.3 cm^−1^. Elemental analysis (%): Calc. for C_25_H_32_N_4_O_3_ (M = 436.56): C, 68.78; H, 7.39; N, 12.83. Found: C, 69.02; H, 7.10; N, 12.57. mp: 95–98 °C.

#### 3.2.7. (±)-(2. R,4R)-2-Benzoyl-6-(dimethylamino)-4-((2,2-dimethylhydrazono) methyl)-4-methyl-1,2,3,4-tetrahydroquinoline (**2g**)

Prepared from the in situ-generated imine **1e** (0.5 mmol) and (E)-1,1-dimethyl-2-(2-methylallylidene)hydrazine (84 mg, 0.75 mmol). Chromatography: dichloromethane: ethyl acetate 4:1. Yield: 108 mg (59%), as a yellow oil. ^1^H-NMR (250 MHz, CDCl_3_) δ: 8.03–7.93 (m, 2H), 7.69–7.59 (m, 1H), 7.57–7.48 (m, 2H), 6.77 (s, 2H), 6.70–6.62 (m, 1H), 6.60 (s, 1H), 5.12 (dd, *J* = 12.3, 2.6 Hz, 1H), 2.86 (s, 6H), 2.75 (s, 6H), 2.09 (dd, *J* = 12.9, 2.7 Hz, 1H), 1.83–1.66 (m, 4H) ppm. ^13^C-NMR (63 MHz, CDCl_3_) δ: 200.0, 144.4, 144.2, 135.7, 135.1, 133.9, 129.3, 128.7, 128.4, 117.7, 115.8, 115.2, 54.7, 43.8, 42.9, 41.5, 39.5, 26.6 ppm. This compound decomposes rapidly into a complex mixture after the purification, preventing further characterization.

#### 3.2.8. (±)-(2. R,4R)-2-Benzoyl-4-((2,2-dimethylhydrazono)methyl)-4,5,7-trimethyl- 1,2,3,4-tetrahydroquinoline (**2h**)

Prepared from the in situ-generated imine **1f** (0.5 mmol) and (E)-1,1-dimethyl-2-(2-methylallylidene)hydrazine (84 mg, 0.75 mmol). Chromatography: petroleum ether: ethyl acetate 94:6. Yield: 133 mg (76%), as a white solid. ^1^H-NMR (250 MHz, CDCl_3_) δ: 8.00–7.95 (m, 2H), 7.69-7.61 (m, 1H), 7.56–7.50 (m, 2H), 6.63 (s, 1H), 6.50 (s, 1H), 6.40 (s, 1H), 5.06 (dd, *J* = 12.3, 2.5 Hz, 1H), 4.89 (bs, 1H), 2.73 (s, 6H), 2.25 (s, 3H), 2.24 (s, 3H), 1.97 (dd, *J* = 12.8, 2.5 Hz, 1H), 1.76 (s, 3H), 1.68 (t, *J* = 12.5 Hz, 1H) ppm. ^13^C-NMR (63 MHz, CDCl_3_) δ: 199.6, 145.5, 143.8, 137.8, 137.6, 135.0, 134.0, 129.3, 128.7, 122.9, 121.6, 114.9, 54.0, 43.6, 42.1, 41.4, 23.4, 22.3, 21.2 ppm. IR (neat) ν: 3333.7, 2918.9, 1682.9 cm^−1^. Elemental analysis (%): calc. for C_22_H_27_N_3_O (M = 349,48): C, 75.61; H, 7.79; N, 12.02. Found: C, 75.58; H, 8.01; N, 12.21. mp: 153–154 °C.

#### 3.2.9. (±)-(2. R,4R)-2-Benzoyl-4-((2,2-dimethylhydrazono)methyl)-4,7-dimethyl- 1,2,3,4-tetrahydroquinoline (**2i**)

Prepared from the in situ-generated imine **1g** (0.5 mmol) and (E)-1,1-dimethyl-2-(2-methylallylidene)hydrazine (84 mg, 0.75 mmol). Chromatography: petroleum ether: ethyl acetate 85:15. Yield: 69 mg (41%), as a yellow solid. ^1^H-NMR (250 MHz, CDCl_3_) δ: 8.02–7.94 (m, 2H), 7.69–7.59 (m, 1H), 7.57–7.47 (m, 2H), 6.96 (d, *J* = 7.8 Hz, 1H), 6.61 (s, 1H), 6.58–6.52 (m, 2H), 5.14 (dd, *J* = 12.3, 2.7 Hz, 1H), 4.84 (bs, 1H), 2.75 (s, 6H), 2.29 (s, 3H), 2.11 (dd, *J* = 12.9, 2.9 Hz, 1H), 1.75 (t, *J* = 12.6 Hz, 1H), 1.66 (s, 3H) ppm. ^13^C-NMR (63 MHz, CDCl_3_) δ: 199.8, 143.8, 142.8, 137.9, 135.1, 134.0, 129.3, 128.7, 128.3, 124.2, 119.1, 116.4, 54.5, 43.7, 40.9, 39.1, 26.5, 21.6 ppm. IR (neat) ν: 3392.6, 2951.7, 1685.4, 1594.4 cm^−1^. Elemental analysis (%): Calc. for C_21_H_25_N_3_O (M = 335.44): C, 75.19; H, 7.51; N, 12.53. Found: C, 75.03; H, 7.27; N, 12.86. mp: 111–113 °C.

#### 3.2.10. (±)-(2. R,4R)-2-Benzoyl-4-((2,2-dimethylhydrazono)methyl)-6-methoxy-4- methyl-1,2,3,4-tetrahydroquinoline (2j)

Prepared from the in situ-generated imine 1h (0.5 mmol) and (E)-1,1-dimethyl-2-(2-methylallylidene)hydrazine (84 mg, 0.75 mmol). Chromatography: petroleum ether: ethyl acetate 9:1. Yield: 116 mg (61%), as a yellow solid. Characterization data were identical to those described in the literature [13].

#### 3.2.11. (±)-(2. R,4R)-4-((2,2-Dimethylhydrazono)methyl)-4,6-dimethyl-2-(4-methoxy- benzoyl)-1,2,3,4-tetrahydroquinoline (**2k**)

Prepared from the in situ-generated imine **1i** (0.5 mmol) and (E)-1,1-dimethyl-2-(2-methylallylidene)hydrazine (84 mg, 0.75 mmol). Chromatography: petroleum ether: ethyl acetate 95:5. Yield: 130 mg (71%), as a yellow solid. ^1^H-NMR (250 MHz, CDCl_3_) δ: 7.96 (d, *J* = 8.7 Hz, 2H), 7.15–6.78 (m, 4H), 6.68 (d, *J* = 8.0 Hz, 1H), 6.56 (s, 1H), 5.06 (d, *J* = 11.7 Hz, 1H), 4.71 (bs, 1H), 3.89 (s, 3H), 2.75 (s, 6H), 2.23 (s, 3H), 2.07 (dd, *J* = 13.0, 2.8 Hz, 1H), 1.74 (t, *J* = 12.8 Hz, 1H), 1.64 (s, 3H) ppm. ^13^C-NMR (63 MHz, CDCl_3_) δ: 197.9, 163.9, 143.6, 140.4, 130.7, 128.4, 127.6, 126.9, 126.8, 116.0, 114.1, 55.6, 53.8, 43.4, 40.9, 39.3, 26.3, 20.6 ppm. IR (neat) ν: 3356.2, 2961.2, 1668.2, 1601.3 cm^-1^. Elemental analysis (%): Calc. for C_22_H_27_N_3_O_2_ (M = 365.48): C, 72.30; H, 7.45; N, 11.50. Found: C, 72.17; H, 7.31; N, 11.27. mp: 118-121 °C.

#### 3.2.12. (±)-(2. R,4R)-4-((2,2-Dimethylhydrazineylidene)methyl)-2-(4- methoxybenzoyl)-4,6,8-trimethyl-1,2,3,4-tetrahydroquinoline (**2l**)

Prepared from the in situ-generated imine **1j** (0.5 mmol) and (E)-1,1-dimethyl-2-(2-methylallylidene)hydra- zine (84 mg, 0.75 mmol). Chromatography: petroleum ether: ethyl acetate 95:5. Yield: 188 mg (99 %), as a yellow solid. ^1^H NMR (250 MHz, CDCl_3_) δ: 8.05–7.91 (m, 2H), 7.01–6.95 (m, 2H), 6.83 (s, 1H), 6.76 (s, 1H), 6.57 (s, 1H), 5.10 (dd, *J* = 12.5, 2.8 Hz, 1H), 4.55 (bs, 1H), 3.90 (s, 3H), 2.74 (s, 6H), 2.21 (s, 6H), 2.07 (dd, *J* = 13.0, 2.8 Hz, 1H), 1.70 (t, *J* = 12.7 Hz, 1H), 1.66 (s, 3H) ppm. ^13^C NMR (63 MHz, CDCl_3_) δ: 198.0, 163.9, 144.2, 138.4, 130.8, 129.7, 127.6, 126.4, 126.3, 126.2, 123.5, 114.2, 55.7, 53.9, 43.5, 41.0, 39.3, 26.2, 20.6, 17.6 ppm. IR (neat) ν: 3365.9, 2954.7, 1669.2, 1611.2 cm^−1^. Elemental analysis (%): Calc. for C_23_H_29_N_3_O_2_ (M = 379.50): C, 72.79; H, 7.70; N, 11.07. Found: C, 72.51; H, 7.34; N, 11.36. mp: 137–140 °C.

#### 3.2.13. (±)-(2. R,4R)-4-((2,2-Dimethylhydrazono)methyl)-2-(4-fluorobenzoyl)-6- methoxy-4-methyl-1,2,3,4-tetrahydroquinoline (**2m**)

Prepared from the in situ-generated imine **1k** (0.5 mmol) and (*E*)-1,1-dimethyl-2-(2-methylallylidene)hydra- zine (84 mg, 0.75 mmol). Chromatography: petroleum ether: ethyl acetate 92:8. Yield: 172 mg (93%), as a yellow solid. Characterization data were identical to those described in the literature. [13]

#### 3.2.14. (±)-(2. R,4R)-4-((2,2-Dimethylhydrazono)methyl)-4-ethyl-2- (4-fluorobenzoyl)6,8-dimethyl-1,2,3,4-tetrahydroquinoline (**2n**)

Prepared from the in situ-generated imine **1l** (0.5 mmol) and (*E*)-1,1-dimethyl-2-(2-methylenebutylidene)hy- drazine (95 mg, 0.75 mmol). Chromatography: petroleum ether: ethyl acetate 95:5. Yield: 189 mg (99%), as a viscous yellow liquid. ^1^H-NMR (250 MHz, CDCl_3_) δ: 8.12–7.85 (m, 2H), 7.24–7.10 (m, 2H), 6.83 (s, 1H), 6.74 (s, 1H), 6.55 (s, 1H), 5.07 (d, *J* = 11.4 Hz, 1H), 4.53 (bs, 1H), 2.71 (s, 6H), 2.22–2.17 (m, 7H), 2.05 (q, *J* = 7.3 Hz, 2H), 1.68 (t, *J* = 12.9 Hz, 1H), 1.01 (t, *J* = 7.3 Hz, 3H) ppm. ^13^C-NMR (63 MHz, CDCl_3_) δ: 198.8, 166.0 (d, *J* = 252 Hz), 142.0, 138.2, 131.4 (d, *J* = 2.9 Hz), 131.0 (d, *J* = 9 Hz), 129.7, 126.6, 125.9, 125.5, 123.1, 116.2 (d, *J* = 25.2 Hz), 54.1, 43.7, 43.4, 35.2, 30.7, 20.7, 17.8, 9.3 ppm. IR (neat) ν: 3370.9, 2964.5, 1673.1, 1603.3 cm^−1^. Elemental analysis (%): Calc. for C_23_H_28_FN_3_O (M = 381.50): C, 72.41; H, 7.40; N, 11.01. Found: C, 72.10; H, 7.10; N, 10.57.

#### 3.2.15. (±)-(2. R,4R)-4-((2,2-Dimethylhydrazono)methyl)-6-methoxy-4-methyl-2- (4-methylbenzoyl)-1,2,3,4-tetrahydro-1,5-naphthyridine (**2o**)

Prepared from the in situ-generated imine **1m** (0.5 mmol) and (*E*)-1,1-dimethyl-2- (2-methylallylidene)hydrazine (84 mg, 0.75 mmol). Chromatography: petroleum ether: ethyl acetate 8:1. Yield: 156 mg (85%), as a yellow solid. ^1^H-NMR (300 MHz, CDCl_3_) δ: 7.90 (d, *J* = 8.3 Hz, 2H), 7.33 (d, *J* = 7.9 Hz, 2H), 7.20 (bs, 1H), 7.07 (d, *J* = 8.6 Hz, 1H), 6.53 (d, *J* = 8.6 Hz, 1H), 5.04 (dd, *J* = 12.2, 2.8 Hz, 1H), 3.86 (s, 3H), 2.70 (s, 6H), 2.46 (s, 3H), 2.28 (dd, *J* = 13.5, 2.8 Hz, 1H), 2.09–1.94 (m, 1H), 1.68 (s, 3H) ppm. ^13^C-NMR (75 MHz, CDCl_3_) δ: 199.5, 157.2, 145.0, 144.7, 143.5, 132.9, 132.5, 130.0, 129.0, 128.7, 109.5, 54.6, 53.6, 43.7, 42.2, 38.1, 27.2, 22.1 ppm. IR (neat) ν: 3316.1, 2960.5, 1676.8, 1605.5 cm^−1^. Elemental analysis (%): Calc. for C_21_H_26_N_4_O_2_ (M = 366.46): C, 68.83; H, 7.15; N, 15.29. Found: C, 68.62; H, 6.80; N, 14.90. mp: 123–125 °C.

#### 3.2.16. (±)-(2. R,4R)-2-(4-Chlorobenzoyl)-4-((2,2-dimethylhydrazono)methyl)- 4,8-dimethyl-1,2,3,4-tetrahydroquinoline (**2p**)

Prepared from the in situ-generated imine **1n** (0.5 mmol) and (*E*)-1,1-dimethyl-2-(2-methylallylidene)hydrazine (84 mg, 0.75 mmol). Chromatography: petroleum ether: ethyl acetate 95:5. Yield: 110 mg (60%), as a pale yellow solid. ^1^H-NMR (300 MHz, CDCl_3_) δ: 8.00–7.88 (m, 2H), 7.59–7.46 (m, 2H), 7.06–6.93 (m, 2H), 6.68 (t, *J* = 7.5 Hz, 1H), 6.59 (bs, 1H), 5.13 (dd, *J* = 12.3, 2.5 Hz, 1H), 4.69 (s, 1H), 2.77 (s, 6H), 2.27 (s, 3H), 2.08 (dd, *J* = 12.9, 3.0 Hz, 1H), 1.76 (t, *J* = 12.7 Hz, 1H), 1.67 (s, 3H) ppm. ^13^C-NMR (75 MHz, CDCl_3_) δ: 198.8, 143.7, 140.8, 140.5, 133.4, 130.2, 129.7, 129.1, 126.6, 126.3, 123.3, 117.6, 54.7, 43.7, 41.3, 39.0, 26.7, 18.0 ppm. IR (neat) ν: 3425.7, 2946.1, 1684.9, 1591.8 cm^−1^. Elemental analysis (%): Calc. for C_21_H_24_ClN_3_O (M = 369.89): C, 68.19; H, 6.54; N, 11.36. Found: C, 68.23; H, 6.22; N, 11.22. mp: 153–156 °C.

#### 3.2.17. (±)-(2. R,4R)-2-(3,4-Dichlorobenzoyl)-4-((2,2-dimethylhydrazono)methyl)- 6-methoxy-4-methyl-1,2,3,4-tetrahydroquinoline (**2q**)

Prepared from the in situ-generated imine **1o** (0.5 mmol) and (E)-1,1-dimethyl-2-(2-methylallylidene)hydra- zine (84 mg, 0.75 mmol). Chromatography: petroleum ether: ethyl acetate 9:1. Yield: 170 mg (81%), as a yellow solid. Characterization data were identical to those described in the literature [37].

#### 3.2.18. (±)-(2. R,4R)-4-((2,2-Dimethylhydrazono)methyl)-2-(2-furylcarbonyl)- 6-methoxy-4-methyl-1,2,3,4-tetrahydroquinoline (**2r**)

Prepared from the in situ-generated imine **1p** (0.5 mmol) and (E)-1,1-dimethyl-2-(2-methylallylidene)hydra- zine (84 mg, 0.75 mmol). Chromatography: petroleum ether: ethyl acetate 85:15. Yield: 145 mg (85%), as a yellow solid. Characterization data were identical to those described in the literature [13].

#### 3.2.19. (±)-(2. R,4R)-4-((2,2-Dimethylhydrazono)methyl)-6-methoxy-4-methyl-2- (thiophen-2-ylcarbonyl)-1,2,3,4-tetrahydroquinoline (**2s**)

Prepared from the in situ-generated imine **1q** (0.5 mmol) and (*E*)-1,1-dimethyl-2-(2-methylallylidene)hydra- zine (84 mg, 0.75 mmol). Chromatography: petroleum ether: ethyl acetate 8:1. Yield: 129 mg (72%), as a pale brown solid. ^1^H-NMR (250 MHz, CDCl_3_) δ: 7.82 (dd, *J* = 3.8, 1.1 Hz, 1H), 7.68 (dd, *J* = 5.0, 1.1 Hz, 1H), 7.17 (dd, *J* = 4.9, 3.8 Hz, 1H), 6.71–6.68 (m, 2H), 6.65 (m, 1H), 6.56 (s, 1H), 4.90 (dd, *J* = 12.1, 3.0 Hz, 1H), 3.73 (s, 3H), 2.73 (s, 6H), 2.14 (dd, *J* = 13.0, 3.1 Hz, 1H), 1.92 (t, *J* = 12.4 Hz, 1H), 1.62 (s, 3H) ppm. ^13^C-NMR (63 MHz, CDCl_3_) δ: 192.9, 152.6, 143.2, 141.1, 136.6, 134.4, 132.7, 128.6, 117.3, 113.9, 56.1, 56.0, 43.6, 41.2, 40.2, 26.8 ppm. IR (neat) ν: 3367.8, 2957.3, 1661.8, 1597.0 cm^−1^. Elemental analysis (%): Calc. for C_19_H_23_N_3_O_2_S (M = 357.47): C, 63.84; H, 6.49; N, 11.75; S, 8.97. Found: C, 63.65; H, 6.22; N, 11.59; S, 8.92. mp: 107–108 °C.

#### 3.2.20. (±)-(2. R,4R)-Ethyl 4-((E)-(2,2-dimethylhydrazono)methyl)-6-methoxy- 4-methyl-1,2,3,4-tetrahydroquinoline-2-carboxylate (**2t**)

Prepared from the in situ-generated imine **1r** (0.5 mmol) and (*E*)-1,1-dimethyl-2-(2-methylallylidene)hydra- zine (84 mg, 0.75 mmol). Chromatography: petroleum ether: ethyl acetate 8:1. Yield: 112 mg (70%), as a pale yellow viscous liquid. Characterization data were identical to those described in the literature [11].

#### 3.2.21. (±)-(2. R,4R)-Ethyl 4-((2,2-dimethylhydrazono)methyl)-4-ethyl-6-methoxy- 1,2,3,4-tetrahydroquinoline-2-carboxylate (**2u**)

Prepared from the in situ-generated imine **1r** (0.5 mmol) and (*E*)-1,1-dimethyl-2-(2-methylenebutylidene)hydrazine (95 mg, 0.75 mmol). Chromatography: petroleum ether: ethyl acetate 9:1. Yield: 107 mg (64%), as an orange oil. Characterization data were identical to those described in the literature [13].

### 3.3. 6-Methoxy-4-methyl-1,5-naphthyridin-2-yl)(phenyl)methanone (**3d**)

Tetrahydronaphthyridine **2d** (100 mg, 0.284 mmol) and 2,3-dichloro-5,6-dicyano- 1,4-benzoquinone (129 mg, 0.567 mmol) were added to a 25 mL zirconia milling jar with a single zirconia ball 20 mm in diameter. The vessel was fixed to the horizontal arm of a mixer mill and it was shaken for 60 min at a frequency of 20 Hz. The resulting paste was recovered from the vessel by washing with dichlorometane, which was subsequently removed under reduced pressure. A silica gel flash chromathography using petroleum ether: ethyl acetate 8:2 as the mobile phase was performed to obtain 75 mg (95%) of **3d** as a white solid. ^1^H-NMR (250 MHz, CDCl_3_) δ: 8.32 (d, *J* = 9.1 Hz, 1H), 8.24–8.12 (m, 3H), 7.70–7.60 (m, 1H), 7.58–7.47 (m, 2H), 7.20 (d, *J* = 9.1 Hz, 1H), 4.16 (s, 3H), 2.83 (d, *J* = 0.5 Hz, 3H) ppm. ^13^C-NMR (63 MHz, CDCl_3_) δ: 193.9, 163.2, 151.9, 146.6, 143.0, 141.1, 139.9, 136.7, 133.4, 131.8, 128.6, 125.2, 117.7, 54.4, 17.7 ppm. IR (neat) ν: 2942.9, 1161.7, 1608.9 cm^-1^. Elemental analysis (%): Calc. for C_17_H_14_N_2_O_2_ (M = 278.31): C, 73.37; H, 5.07; N, 10.07. Found: C, 73.66; H, 5.26; N, 10.05,. mp: 121–123 °C.

## 4. Conclusions

Mechanochemical activation by vibratory ball milling (20 Hz, ZrO_2_ ball and milling jar) was shown to promote the sequential three-component aza-vinylogous Povarov reaction between aromatic amines, α-ketoaldehydes or α-formylesters and α,β-unsaturated dimethylhydrazones to furnish 1,2,3,4-tetrahydroquinolines and 1,2,3,4-tetrahydro-1,5-naphthyridines bearing two or three functional groups, respectively. Our results show that the mechanochemical protocol, besides having the advantages associates to one-pot operation, leads to much faster reactions, in comparable yields and slightly lower diastereoselectivities in comparison with solution chemistry. The time saved on average is 1–2 h for ball-milling vs solution chemistry at room temperature, although there are examples of a 4 h time saving. A combination of this aza-Povarov reaction, using 6-methoxypyridin-3-amine as a starting material, with DDQ treatment afforded 2-acyl-1,5-naphthyridines in a fully mechanochemical fashion.

## Figures and Tables

**Table 1:** Optimization of the synthesis of compound **2a**.

Entry	Catalyst (eqs.)	Yield (%)	Dr (*cis*:*trans*)	Milling Type (Frequency)	Jar Material ^a^
1	−	31	67:33	HSVM (20 Hz)	ZrO_2_
2	CAN (0.1)	54	69:31	HSVM (20 Hz)	ZrO_2_
3	FeCl_3_ (0.1)	51	70:30	HSVM (20 Hz)	ZrO_2_
4	AlCl_3_ (0.1)	49	72:28	HSVM (20 Hz)	ZrO_2_
5	Sc(TfO)_3_ (0.1)	72	73:27	HSVM (20 Hz)	ZrO_2_
6	Yb(TfO)_3_ (0.1)	73	78:22	HSVM (20 Hz)	ZrO_2_
7	Eu(hfc)_3_ (0.05)	35	55:45	HSVM (20 Hz)	ZrO_2_
8	BF_3_ ·Et_2_O (0.1)	69	75:25	HSVM (20 Hz)	ZrO_2_
9	ZnCl_2_ (0.1)	60	75:25	HSVM (20 Hz)	ZrO_2_
10	InCl_3_ (0.1)	90	71:29	HSVM (20 Hz)	ZrO_2_
11	InCl_3_ (0.2)	78	69:31	HSVM (20 Hz)	ZrO_2_
12	InCl_3_ (0.1)	72	71:29	HSVM (20 Hz)	ZrO_2_^b^
13	InCl_3_ (0.1)	67	71:29	HSVM (25 Hz)	ZrO_2_
14	InCl_3_ (0.1)	45	71:29	HSVM (10 Hz)	Stainless steel ^c^
15	InCl_3_ (0.1)	30	71:29	HSVM (20 Hz)	Stainless steel ^d^
16	InCl_3_ (0.1)	16	71:29	HSVM (30 Hz)	Stainless steel ^d^
17	InCl_3_ (0.1)	22	71:29	PBM (400 rpm)	Stainless steel ^e^
18	InCl_3_ (0.1)	48	71:29	PBM (600 rpm)	Stainless steel ^e^
19	(±)-CSA (0.1)	67	70:30	HSVM (20 Hz)	ZrO_2_
20	*p*-TsOH (0.1)	90	75:25	HSVM (20 Hz)	ZrO_2_
21	*p*-TsOH (0.1)	67 ^f^	75:25	HSVM (20 Hz)	ZrO_2_
22	*p*-TsOH (0.1)	54 ^g^	75:25	HSVM (20 Hz)	ZrO_2_
23	*p*-TsOH (1)	87	75:25	HSVM (20 Hz)	ZrO_2_
24	*p*-TsOH (0.1)	81^h^	75:25	HSVM (20 Hz)	ZrO_2_
25	*p*-TsOH (0.1)	56 ^i^	75:25	HSVM (20 Hz)	ZrO_2_

^a^ Unless specified otherwise, 1 ball of ZrO_2_ 20 mm in diameter was employed and the reaction time for the cycloaddition step was 60 min. ^b^ 1 ball 16 mm in diameter. ^c^ 2 balls 15 mm in diameter were employed. ^d^ 9 balls 6 mm in diameter were employed. ^e^ 25 balls 6 mm in diameter were employed. ^f^ Reaction time, 45 min. ^g^ Reaction time, 90 min. The lower yield was ascribed due to partial decomposition of **2**. Although we were not able to isolate the side products arising from this decomposition process, the crude reaction mixtures seem to show mixtures of **2** and the corresponding 1,4-dihydro- and 3,4-dihydroquinolines arising from its dehydrogenation. ^h^ Reaction performed at a 3 mmol scale. ^i^ The reaction mixture was loaded onto the chromatographic column without workup.

**Table 2:** Comparison of the results of synthesis of 1,2,3,4-tetrahydroquinoline and 1,2,3,4-tetrahydro-1,5-naphthyridine derivatives **2** under mechanochemical and conventional conditions.

Cmpd	Mechanochemical Synthesis			Solution Synthesis ^a^		
	Time, h	Yield, %	Dr, *cis*:*trans*	Time, h	Yield, %	Dr, *cis*:*trans*
**2a**	1	90	75:25	3	72	82:18
**2b**	1	62	75:25	3	63	91:09
**2c**	1	67	86:14	1	72	100:0
**2d**	1	98	86:14	−	−	−
**2e**	1	76	69:31	4	95	98:02
**2f**	1	72	74:26	−	−	−
**2g**	1	59	76:24	−	−	−
**2h**	1	76	55:45	5	50	50:50
**2i**	1	41	72:28	−	−	−
**2j**	1	61	73:27	3	70	83:17
**2k**	1	71	74:26	−	−	−
**2l**	1	99	75:25	−	−	−
**2m**	1	93	72:28	3	75	84:16
**2n**	1	99	75:25	−	−	−
**2o**	1	85	71:29	−	−	−
**2p**	1	60	75:25	−	−	−
**2q**	1	81	82:18	−	−	−
**2r**	1	85	73:27	2	88	100:0
**2s**	1	72	70:30	−	−	−
**2t**	1	70	66:34	−	−	−
**2u**	1	64	75:25	2	74	87:13

^a^ The solution data come from reference [13].

## Data Availability

Please refer to suggested Data Availability Statements in section “MDPI Research Data Policies” at https://www.mdpi.com/ethics.

## Supplementary Materials

The following are available online: ^1^H-NMR data of imines **1** and copies of NMR spectra of new compounds.

## Sample Availability

Not available.
