# Supplementary material for: Mechanochemical Aza-Vinylogous Povarov Reactions for the Synthesis of Highly Functionalized 1,2,3,4-Tetrahydroquinolines and 1,2,3,4-Tetrahydro-1,5-Naphthyridines
Source: Molecules. 2021 Mar 2;26(5):1330. doi: 10.3390/molecules26051330 (PMC7958332; doi:10.3390/molecules26051330)
Supplement: Supplementary file 1 [file molecules-26-01330-s001.pdf]

Supporting information

Mechanochemical aza-vinylogous Povarov reactions for the synthesis of highly functionalized 1,2,3,4-tetrahydroquinolines and 1,2,3,4-tetrahydro- 1,5-naphthyridines

José Clerigué [1], M. Teresa Ramos [1] and J. Carlos Menéndez [1,*]

Unidad de Química Orgánica y Farmacéutica, Departamento de Química en Ciencias Farmacéuticas, Facultad de Farmacia, Universidad Complutense, 28040 Madrid, Spain

TABLE OF CONTENTS

1. ^{1}H-NMR data of *in situ*-generated imines **1** S2

2. Copies of spectra of new compounds S7

1. ^{1}H-NMR data of *in situ*-generated imines 1

(*E*)-2-((4-methoxyphenyl)imino)-1-phenylethanone (1a)

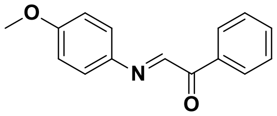

Prepared from *p*-anisidine (62 mg, 0.5 mmol) and phenylglyoxal monohydrate (76 mg, 0.5 mmol).

^{1}H NMR (250 MHz, $CDCl_3$) δ: 8.35 (s, 1H), 8.31 – 8.27 (m, 2H), 7.62 (m, 1H), 7.58 – 7.49 (m, 2H), 7.40 (d, *J* = 9.0 Hz, 2H), 6.96 (d, *J* = 9.0 Hz, 2H), 3.84 (s, 3H) ppm.

(*E*)-2-((6-methoxypyridin-3-yl)imino)-1-phenylethanone (1b)

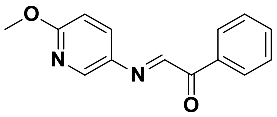

Prepared from 5-amino-2-methoxypyridine (62 mg, 0.5 mmol) and phenylglyoxal monohydrate (76 mg, 0.5 mmol).

^{1}H NMR (250 MHz, $CDCl_3$) δ: 8.38 (s, 1H), 8.33 – 8.23 (m, 3H), 7.73 – 7.60 (m, 2H), 7.57 – 7.47 (m, 2H), 6.89 – 6.80 (m, 1H), 3.99 (s, 3H) ppm.

(*E*)-2-((2,4-dimethylphenyl)imino)-1-phenylethanone (1c)

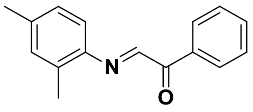

Prepared from 2,4-dimethylaniline (61 mg, 0.5 mmol) and phenylglyoxal monohydrate (76 mg, 0.5 mmol).

^{1}H NMR (250 MHz, $CDCl_3$) δ: 8.36 – 8.33 (m, 2H), 8.23 (s, 1H), 7.61 (m, 1H), 7.54 – 7.47 (m, 2H), 7.13 – 7.03(m, 2H), 7.00 (d, *J* = 8.0 Hz, 1H), 2.40 (s, 3H), 2.36 (s, 3H) ppm.

(*E*)-tert-butyl (4-((2-oxo-2-phenylethylidene)amino)phenyl)carbamate (1d)

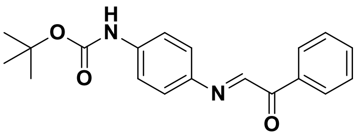

Prepared from *tert*-butyl-(4-aminophenyl)carbamate (104 mg, 0.5 mmol) and phenylglyoxal monohydrate (76 mg, 0.5 mmol).

^{1}H NMR (250 MHz, $CDCl_3$) δ: 8.36 (s, 1H), 8.35 – 8.26 (m, 2H), 7.66 – 7.57 (m, 1H), 7.55 – 7.46 (m, 4H), 7.40 – 7.32 (m, 2H), 7.10 (bs, 1H), 1.53 (s, 9H) ppm.

(*E*)-2-((4-(dimethylamino)phenyl)imino)-1-phenylethanone (1e)

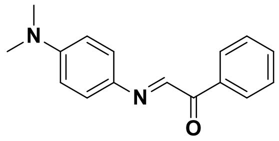

Prepared from *N,N*-dimethyl-*p*-phenylenediamine (68 mg, 0.5 mmol) and phenylglyoxal monohydrate (76 mg, 0.5 mmol).

^{1}H NMR (250 MHz, $CDCl_3$) δ: 8.44 (s, 1H), 8.38 – 8.28 (m, 2H), 7.67 – 7.58 (m, 1H), 7.56 – 7.42 (m, 4H), 6.80 – 6.68 (m, 2H), 3.04 (s, 6H) ppm.

(*E*)-2-((3,5-dimethylphenyl)imino)-1-phenylethanone (1f)

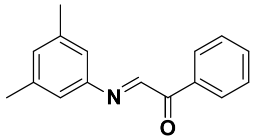

Prepared from 3,5-dimethylaniline (61 mg, 0.5 mmol) and phenylglyoxal monohydrate (76 mg, 0.5 mmol).

^{1}H NMR (250 MHz, $CDCl_3$) δ: 8.34-8.31 (m, 3H), 7.61 (m, 1H), 7.54-7.48 (m, 2H), 7.01-6.98 (m, 3H), 2.38 (s, 6H) ppm.

(*E*)-1-phenyl-2-(*m*-tolylimino)ethenone (1g)

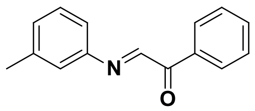

Prepared from *m*-toluidine (54 mg, 0.5 mmol) and phenylglyoxal monohydrate (76 mg, 0.5 mmol).

^{1}H NMR (250 MHz, $CDCl_3$) δ: 8.40-8.32 (m, 3H), 7.70-7.62 (m, 1H), 7.59-7.51 (m, 2H), 7.42-7.34 (m, 1H), 7.25-7.14 (m, 3H), 2.45 (s, 3H) ppm.

(*E*)-1-(4-methoxyphenyl)-2-((4-methoxyphenyl)imino)ethenone (1h)

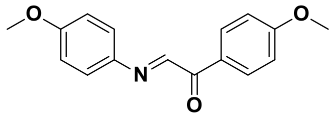

Prepared from *p*-anisidine (62 mg, 0.5 mmol) and *p*-methoxyphenylglyoxal hydrate (99 mg, 0.6 mmol).

^{1}H NMR (250 MHz, $CDCl_3$) δ: 8.35 (d, *J* = 9.1 Hz, 2H), 8.33 (s, 1H), 7.39 (d, *J* = 9.0 Hz, 2H), 6.98 (d, *J* = 9.1 Hz, 2H), 6.96 (d, *J* = 9.0 Hz, 2H), 3.89 (s, 3H), 3.85 (s, 3H) ppm.

(*E*)-1-(4-methoxyphenyl)-2-(p-tolylimino)ethenone (1i)

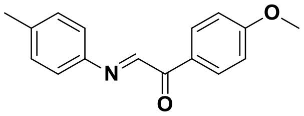

Prepared from *p*-toluidine (54 mg, 0.5 mmol) and *p*-methoxyphenylglyoxal hydrate (99 mg, 0.6 mmol).

^{1}H NMR (250 MHz, MeOD) δ: 8.12 (d, *J* = 9.0 Hz, 2H), 7.00 (d, *J* = 8.8 Hz, 4H), 6.83 (d, *J* = 8.4 Hz, 2H), 6.00 (s, 1H), 3.86 (s, 3H), 2.23 (s, 3H).

(*E*)-2-((2,4-Dimethylphenyl)imino)-1-(4-methoxyphenyl)ethanone (1j)

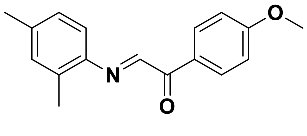

Prepared from 2,4-dimethylaniline (61 mg, 0.5 mmol) and *p*-methoxyphenylglyoxal hydrate (99 mg, 0.6 mmol).

^{1}H-NMR ($CDCl_3$, 250 MHz) δ: 8.47-8.36 (m, 2H), 8.20 (s, 1H), 7.12-7.03 (m, 2H), 7.02-6.94 (m, 2H), 6.97 (d, *J* = 8.0 Hz, 1H), 3.90 (s, 3H), 2.41 (s, 3H), 2.36 (s, 3H) ppm.

(*E*)-1-(4-fluorophenyl)-2-((4-methoxyphenyl)imino)ethenone (1k)

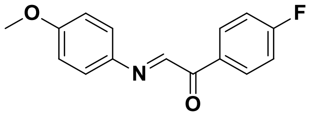

Prepared from *p*-anisidine (62 mg, 0.5 mmol) and *p*-fluorophenylglyoxal (91 mg, 0.6 mmol).

^{1}H NMR (250 MHz, $CDCl_3$) δ: 8.39 (dd, *J* = 9.0, 5.6 Hz, 2H), 8.31 (s, 1H), 7.40 (d, *J* = 9.0 Hz, 2H), 7.17 (t, *J* = 8.8 Hz, 2H), 6.97 (d, *J* = 9.0 Hz, 2H), 3.86 (s, 3H) ppm.

(*E*)-2-((2,4-dimethylphenyl)imino)-1-(4-fluorophenyl)ethenone (1l)

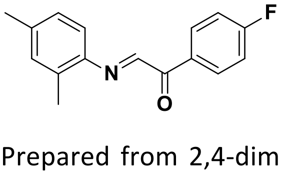

Prepared from 2,4-dimethylaniline (61 mg, 0.5 mmol) and *p*-fluorophenylglyoxal (91 mg, 0.6 mmol).

^{1}H NMR (250 MHz, $CDCl_3$) δ: 8.44 (dd, *J* = 9.0, 5.6 Hz, 2H), 8.20 (s, 1H), 7.18 (t, *J* = 8.7 Hz, 2H), 7.11 (s, 1H), 7.06 (d, *J* = 8.0 Hz, 1H), 7.00 (d, *J* = 8.0 Hz, 1H), 2.40 (s, 3H), 2.36 (s, 3H) ppm.

(*E*)-2-((6-methoxypyridin-3-yl)imino)-1-(*p*-tolyl)ethenone (1m)

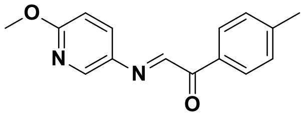

Prepared from 5-amino-2-methoxypyridine (62 mg, 0.5 mmol) and 4-methylphenylglyoxal (89 mg, 0.6 mmol).

^{1}H NMR (250 MHz, $CDCl_3$) δ: 8.37 (s, 1H), 8.28 (d, *J* = 2.6 Hz, 1H), 8.20 (d, *J* = 8.2 Hz, 2H), 7.69 (dd, *J* = 8.9, 2.7 Hz, 1H), 7.32 (d, *J* = 8.1 Hz, 2H), 6.83 (d, *J* = 8.9 Hz, 1H), 3.99 (s, 3H), 2.45 (s, 3H) ppm.

(*E*)-1-(4-chlorophenyl)-2-(*o*-tolylimino)ethanone (1n)

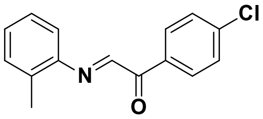

Prepared from *o*-toluidine (54 mg, 0.5 mmol) and 4-chlorophenylglyoxal (101 mg, 0.6 mmol).

^{1}H NMR (250 MHz, $CDCl_3$) δ: 8.41 – 8.32 (m, 2H), 8.21 (s, 1H), 7.54 – 7.48 (m, 2H), 7.34 – 7.26 (m, 3H), 7.11 – 7.04 (m, 1H), 2.45 (s, 3H) ppm.

(*E*)-1-(3,4-dichlorophenyl)-2-((4-methoxyphenyl)imino)ethanone (1o)

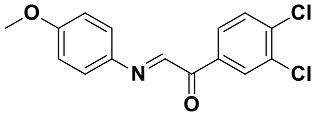

Prepared from *p*-anisidine (62 mg, 0.5 mmol) and 3,4-dichlorophenylglyoxal (122 mg, 0.6 mmol).

^{1}H NMR (250 MHz, DMSO) δ: 8.48 (s, 1H), 8.36 (d, *J* = 1.9 Hz, 1H), 8.13 (dd, *J* = 8.4, 1.9 Hz, 1H), 7.85 (d, *J* = 8.4 Hz, 1H), 7.53 (d, *J* = 8.9 Hz, 2H), 7.05 (d, *J* = 8.9 Hz, 2H), 3.82 (s, 3H) ppm.

(*E*)-1-(furan-2-yl)-2-((4-methoxyphenyl)imino)ethanone (1p)

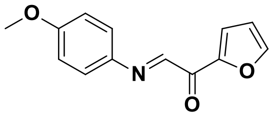

Prepared from *p*-anisidine (62 mg, 0.5 mmol) and 2-furylglyoxal (75 mg, 0.6 mmol).

^{1}H NMR (250 MHz, $CDCl_3$) δ: 8.29 (s, 1H), 8.00 (dd, *J* = 3.5, 0.7 Hz, 1H), 7.78 (dd, *J* = 1.6, 0.7 Hz, 1H), 7.44 (d, *J* = 9.0 Hz, 2H), 7.01 (d, *J* = 9.0 Hz, 2H), 6.65 (dd, *J* = 3.6, 1.7 Hz, 1H), 3.89 (s, 3H) ppm.

(*E*)-2-((4-methoxyphenyl)imino)-1-(thiophen-2-yl)ethenone (1q)

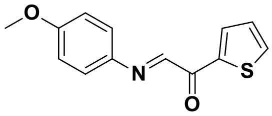

Prepared from *p*-anisidine (62 mg, 0.5 mmol) and 2-thienylglyoxal (84 mg, 0.6 mmol).

^{1}H NMR (250 MHz, $CDCl_3$) δ: 8.36 (dd, *J* = 3.9, 1.2 Hz, 1H), 8.22 (s, 1H), 7.74 (dd, *J* = 5.0, 1.2 Hz, 1H), 7.43 (d, *J* = 9.1 Hz, 2H), 7.18 (dd, *J* = 5.0, 3.9 Hz, 1H), 6.97 (d, *J* = 9.0 Hz, 2H), 3.85 (s, 3H) ppm.

(*E*)-ethyl 2-((4-methoxyphenyl)imino)acetate (1r)

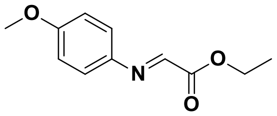

Prepared from *p*-anisidine (62 mg, 0.5 mmol) and ethyl glyoxal monohydrate (77 mg, 0.75 mmol).

^{1}H NMR (250 MHz, $CDCl_3$) δ: 7.89 (s, 1H), 7.32 (d, *J* = 8.9 Hz, 2H), 6.88 (d, *J* = 8.9 Hz, 2H), 4.35 (q, *J* = 7.1 Hz, 2H), 3.77 (s, 3H), 1.35 (t, *J* = 7.1 Hz, 3H) ppm.

2. Copies of spectra of new compounds

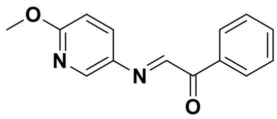

1b

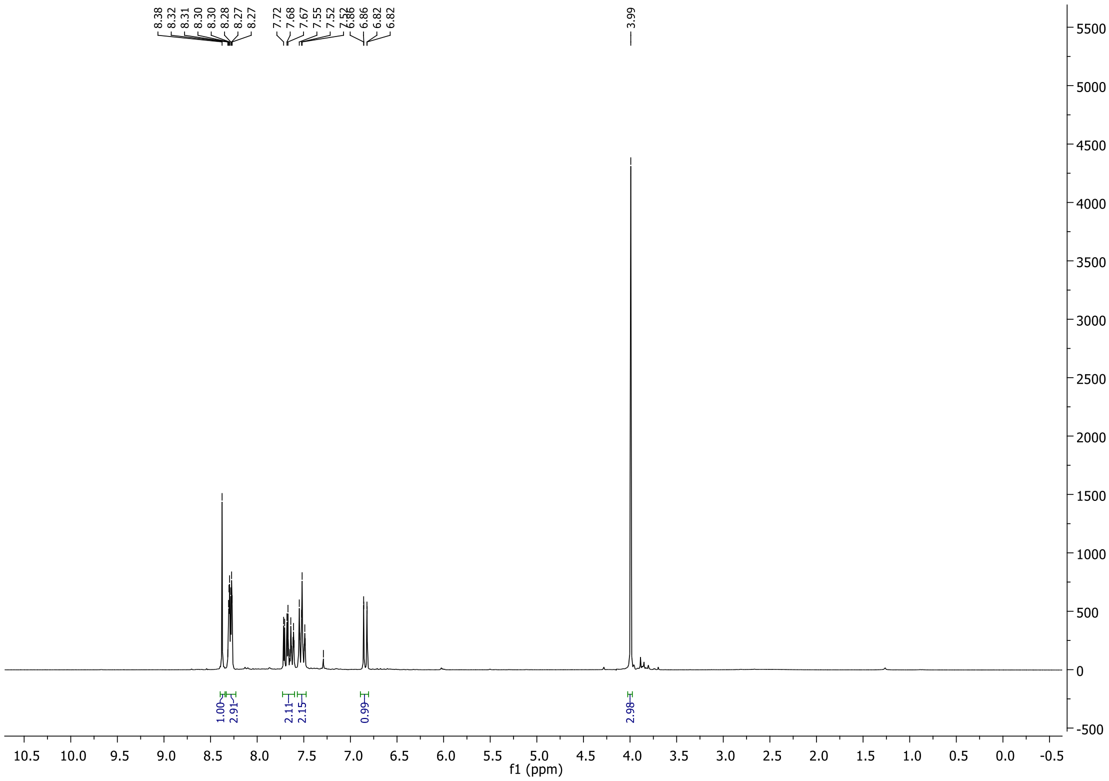

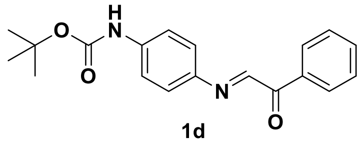
H
N
O
O
N
O

1d

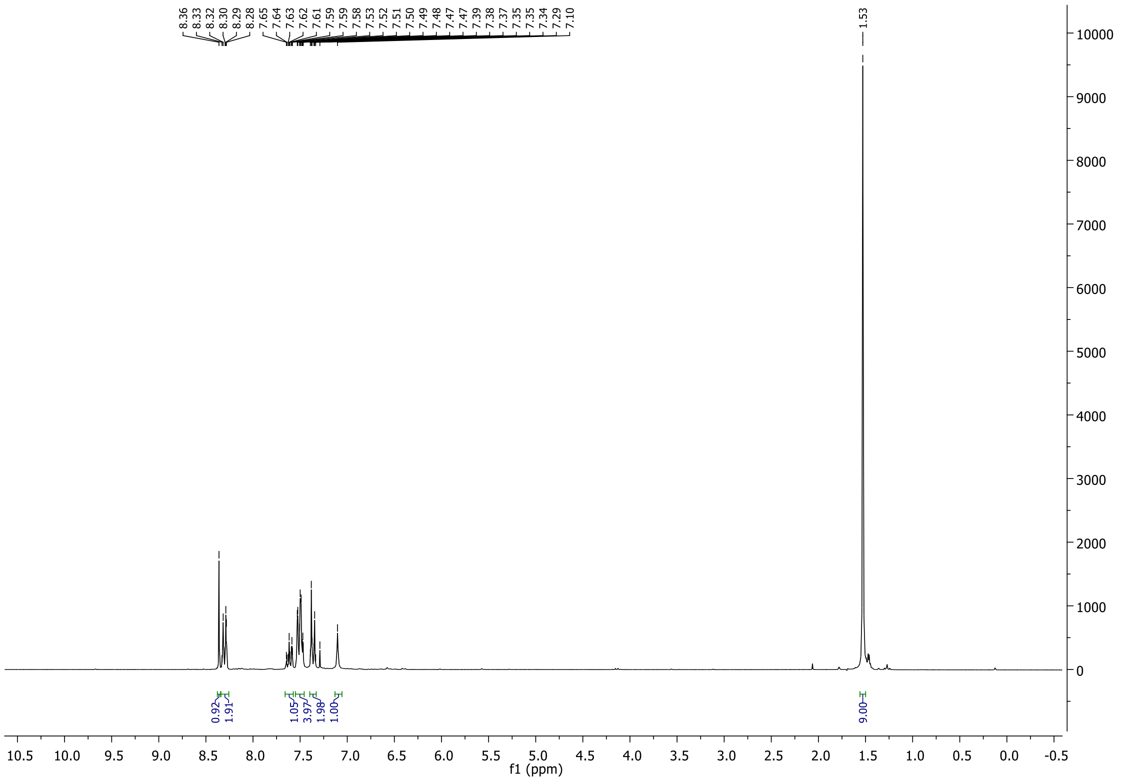
8.36
8.33
8.32
8.30
8.29
8.28
7.65
7.64
7.63
7.62
7.61
7.59
7.59
7.58
7.53
7.52
7.51
7.50
7.49
7.48
7.47
7.39
7.38
7.37
7.35
7.35
7.34
7.29
7.10
1.53
0.92
1.91
1.05
3.97
1.98
1.00
9.00
10000
9000
8000
7000
6000
5000
4000
3000
2000
1000
0
10.5 10.0 9.5 9.0 8.5 8.0 7.5 7.0 6.5 6.0 5.5 5.0 4.5 4.0 3.5 3.0 2.5 2.0 1.5 1.0 0.5 0.0 -0.5
f1 (ppm)

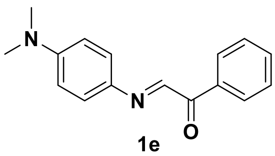

1e

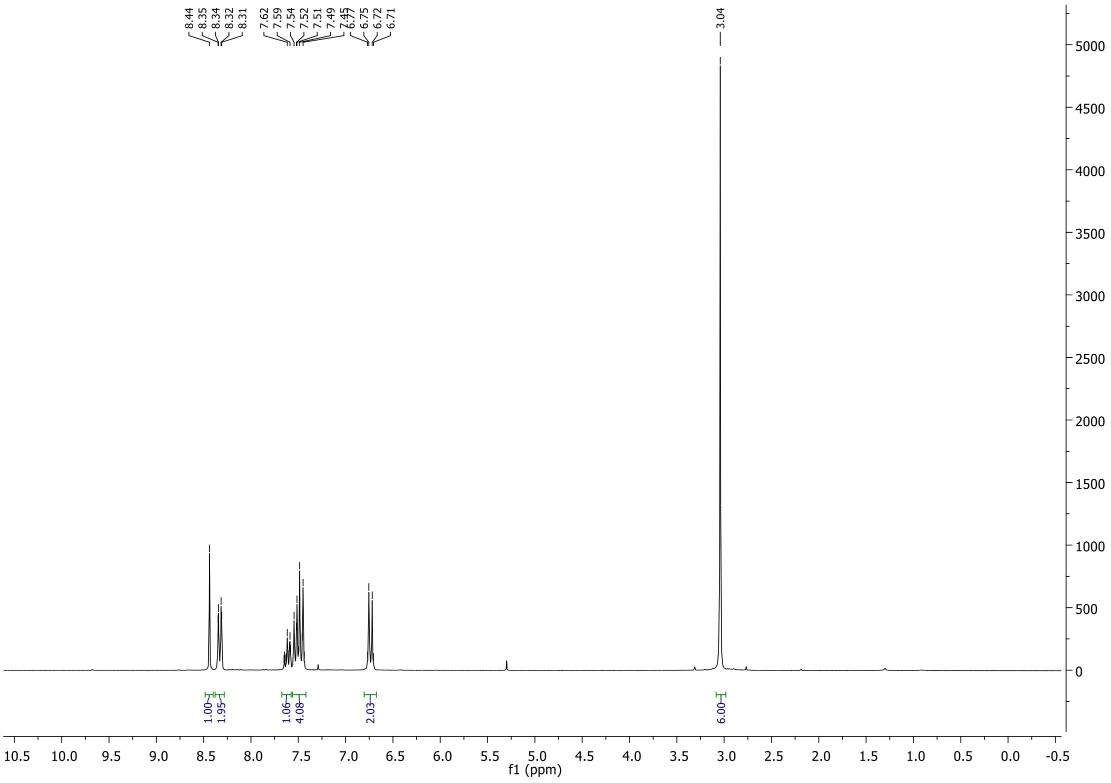

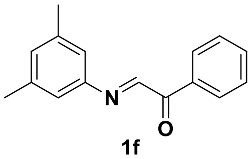
N
O

1f

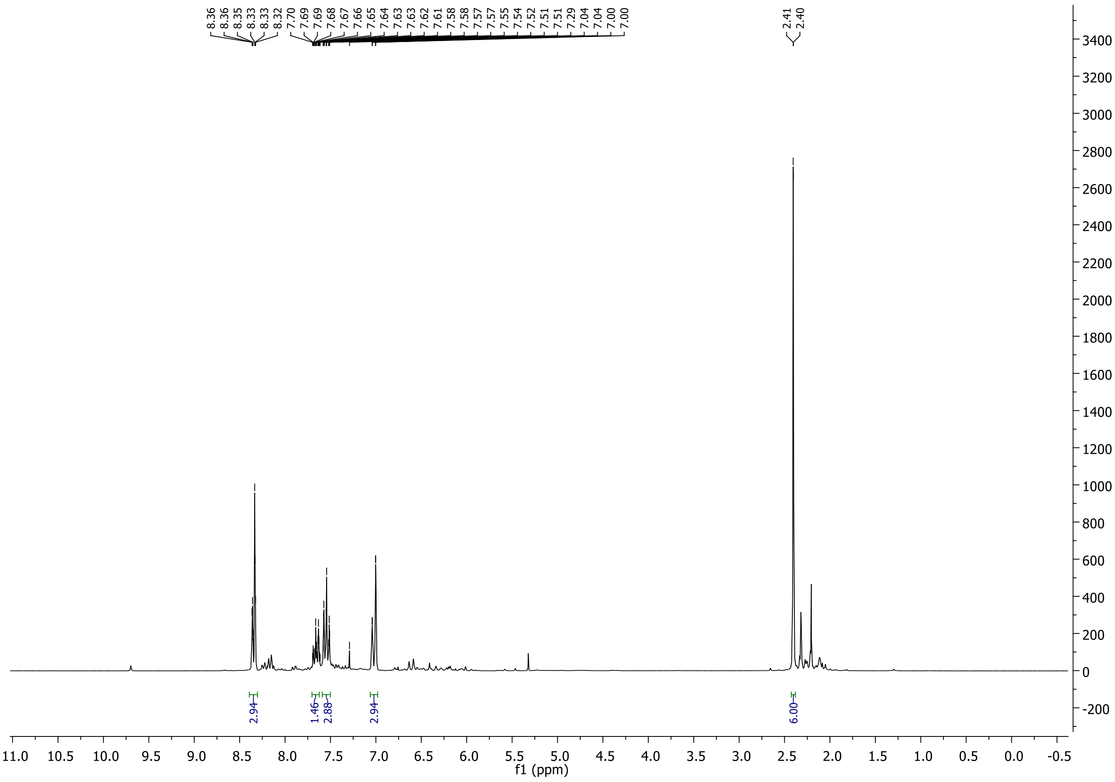
8.36
8.36
8.35
8.33
8.33
8.32
7.70
7.69
7.68
7.67
7.66
7.65
7.64
7.63
7.63
7.62
7.61
7.58
7.58
7.57
7.57
7.55
7.54
7.52
7.51
7.51
7.29
7.04
7.04
7.00
7.00
2.41
2.40
2.94
1.46
2.88
2.94
6.00
11.0 10.5 10.0 9.5 9.0 8.5 8.0 7.5 7.0 6.5 6.0 5.5 5.0 4.5 4.0 3.5 3.0 2.5 2.0 1.5 1.0 0.5 0.0 -0.5
f1 (ppm)
3400 3200 3000 2800 2600 2400 2200 2000 1800 1600 1400 1200 1000 800 600 400 200 0 -200

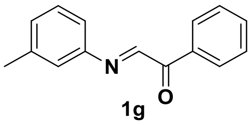
N
O

1g

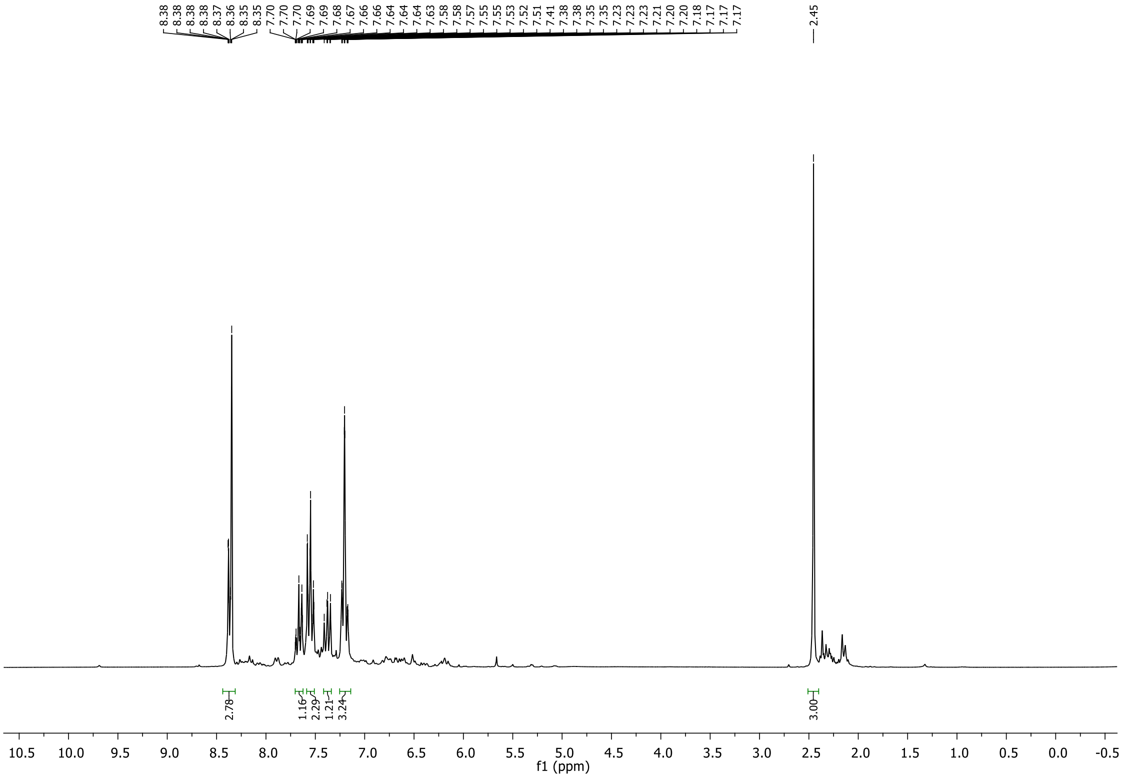
8.38
8.38
8.38
8.38
8.37
8.36
8.35
8.35
7.70
7.70
7.69
7.69
7.68
7.67
7.66
7.66
7.64
7.64
7.64
7.63
7.58
7.58
7.57
7.55
7.55
7.53
7.52
7.51
7.41
7.38
7.38
7.35
7.35
7.23
7.23
7.23
7.21
7.20
7.18
7.17
7.17
7.17
2.45
2.78
1.16
2.29
1.21
3.24
3.00
10.5 10.0 9.5 9.0 8.5 8.0 7.5 7.0 6.5 6.0 5.5 5.0 4.5 4.0 3.5 3.0 2.5 2.0 1.5 1.0 0.5 0.0 -0.5
f1 (ppm)

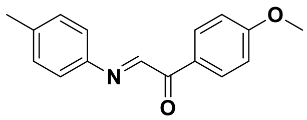
O
N
O

1i

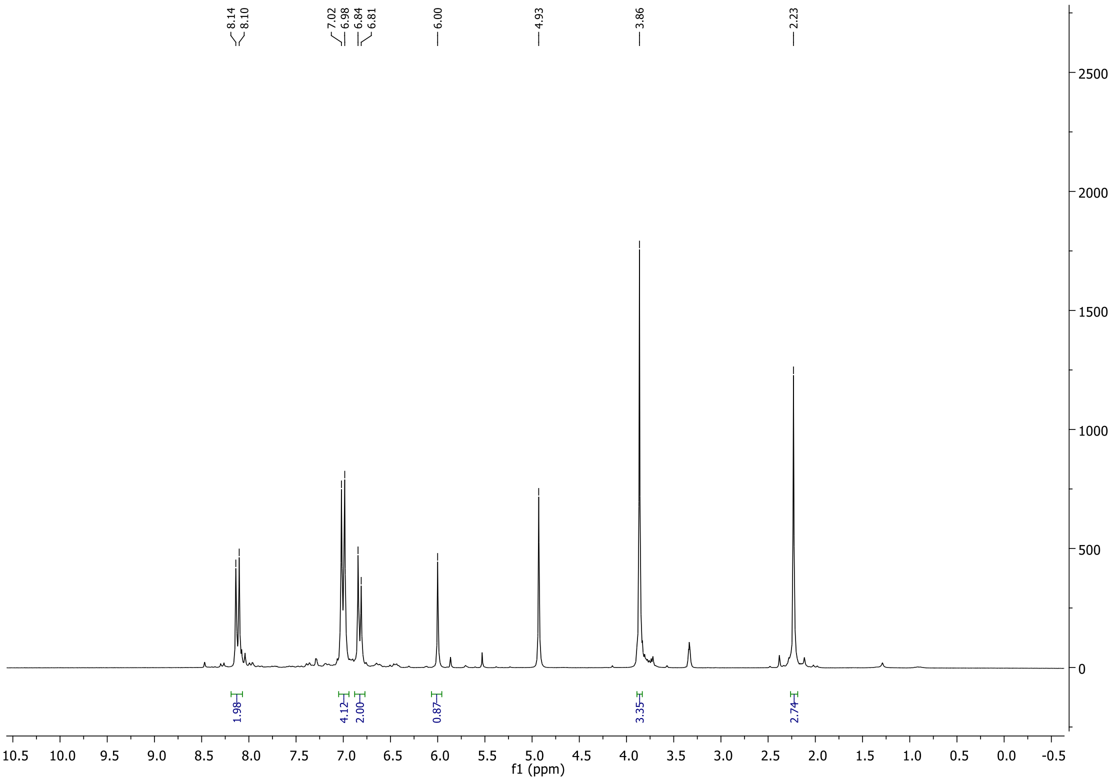
8.14
8.10
7.02
6.98
6.84
6.81
6.00
4.93
3.86
2.23
2500
2000
1500
1000
500
0
1.98
4.12
2.00
0.87
3.35
2.74
10.5 10.0 9.5 9.0 8.5 8.0 7.5 7.0 6.5 6.0 5.5 5.0 4.5 4.0 3.5 3.0 2.5 2.0 1.5 1.0 0.5 0.0 -0.5
f1 (ppm)

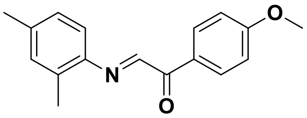

1j

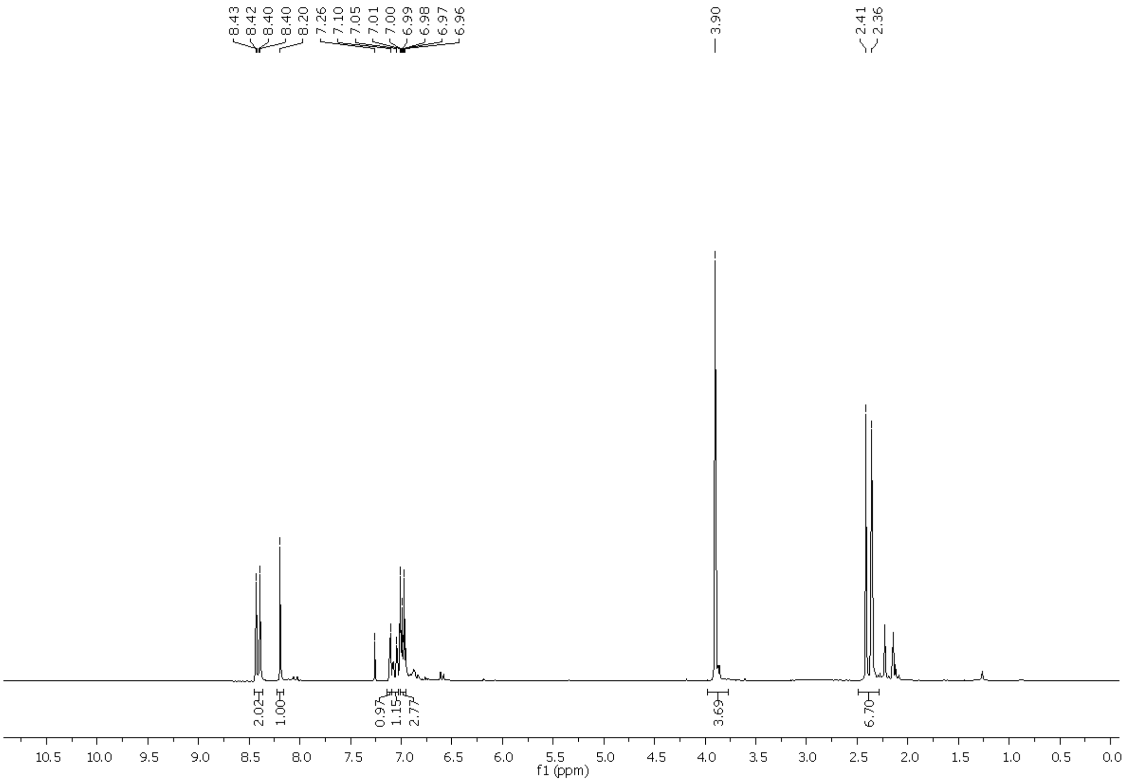
8.43
8.42
8.40
8.40
8.20
7.26
7.10
7.05
7.01
7.00
6.99
6.98
6.97
6.96
3.90
2.41
2.36
2.02
1.00
0.97
1.15
2.77
3.69
6.70
10.5 10.0 9.5 9.0 8.5 8.0 7.5 7.0 6.5 6.0 5.5 5.0 4.5 4.0 3.5 3.0 2.5 2.0 1.5 1.0 0.5 0.0
f1 (ppm)

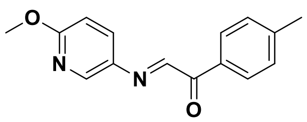

1m

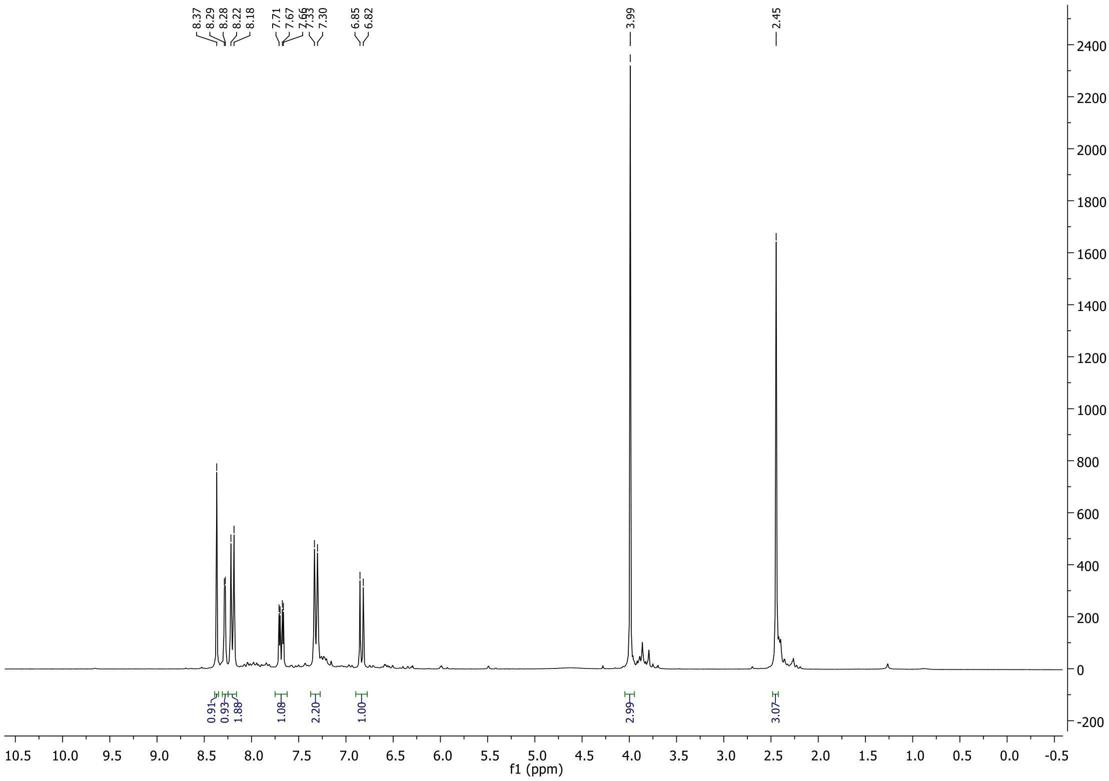
8.37
8.29
8.28
8.22
8.18
7.71
7.67
7.33
7.30
6.85
6.82
3.99
2.45
0.91
0.93
1.88
1.08
2.20
1.00
2.99
3.07
f1 (ppm)

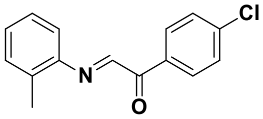
Cl
N
O

1n

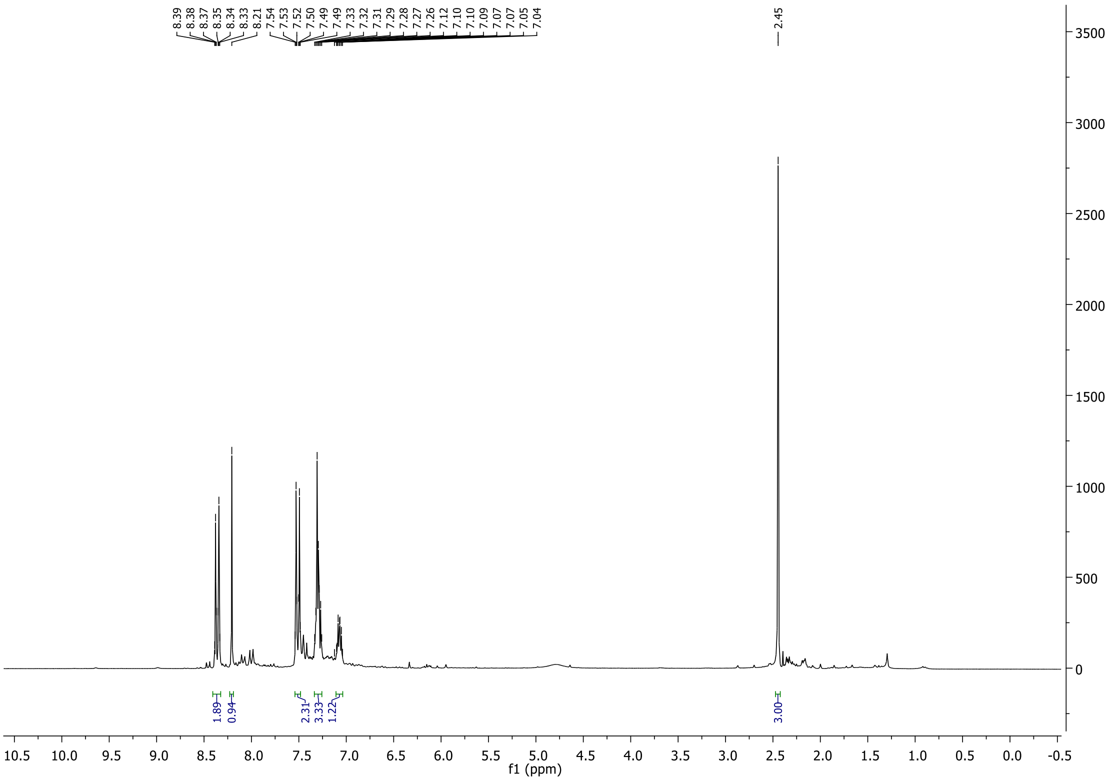
8.39
8.38
8.37
8.35
8.34
8.33
8.21
7.54
7.53
7.52
7.50
7.49
7.49
7.33
7.32
7.31
7.29
7.28
7.27
7.26
7.12
7.10
7.10
7.09
7.07
7.07
7.05
7.04
2.45
1.89
0.94
2.31
3.33
1.22
3.00
3500
3000
2500
2000
1500
1000
500
0
10.5 10.0 9.5 9.0 8.5 8.0 7.5 7.0 6.5 6.0 5.5 5.0 4.5 4.0 3.5 3.0 2.5 2.0 1.5 1.0 0.5 0.0 -0.5
f1 (ppm)

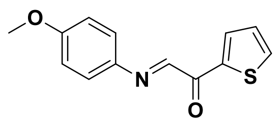

1q

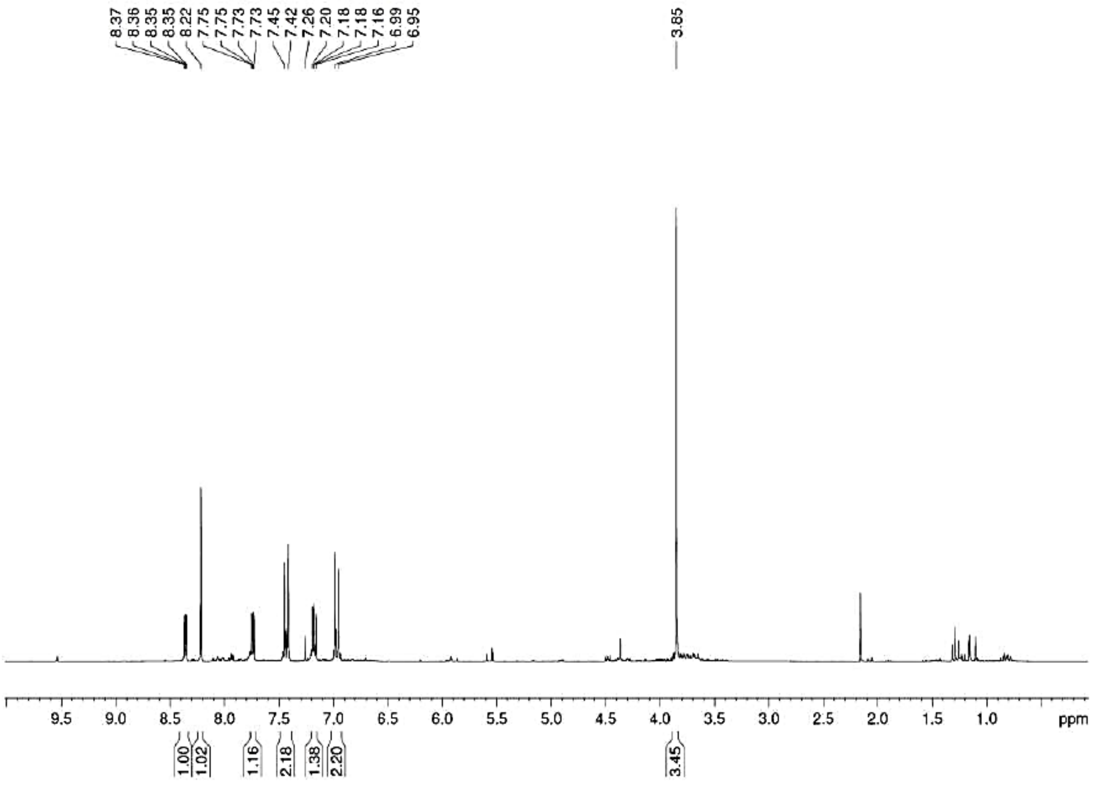
8.37
8.36
8.35
8.35
8.22
7.75
7.75
7.73
7.73
7.45
7.42
7.26
7.20
7.18
7.18
7.16
6.99
6.95
3.85
9.5 9.0 8.5 8.0 7.5 7.0 6.5 6.0 5.5 5.0 4.5 4.0 3.5 3.0 2.5 2.0 1.5 1.0 ppm
1.00
1.02
1.16
2.18
1.38
2.20
3.45

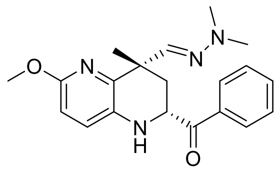
O
N
N
N
N
H
O

2d

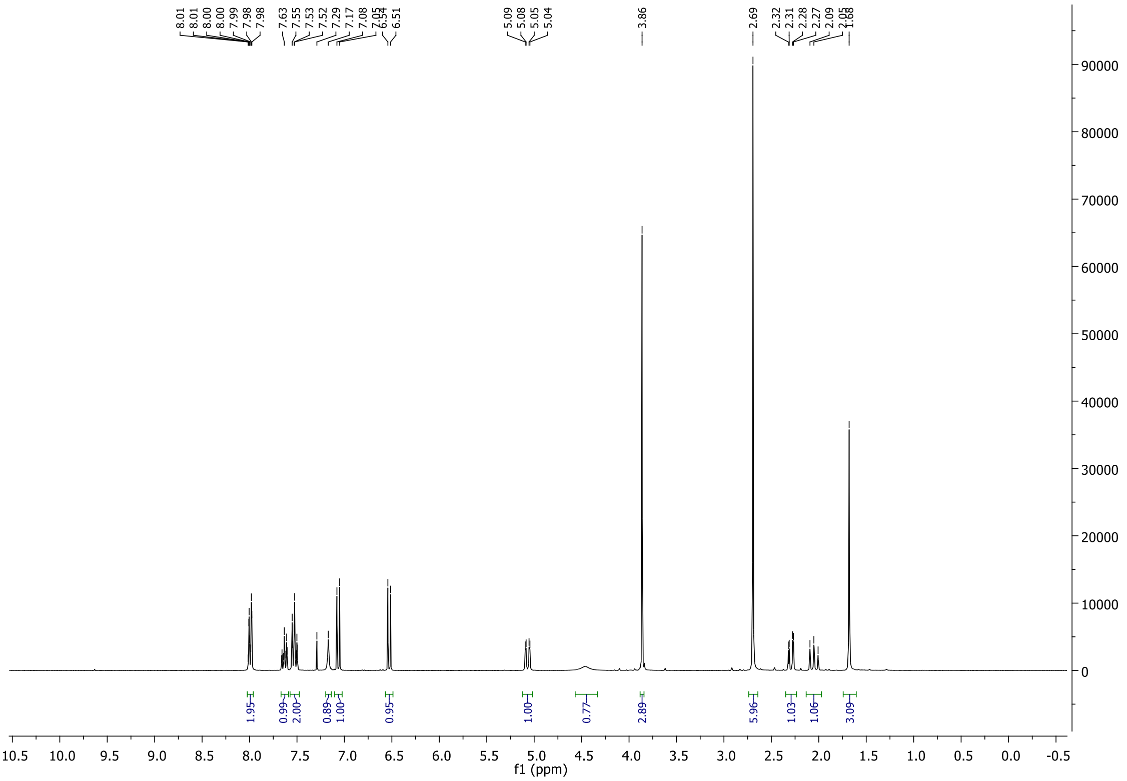
8.01 8.01 8.00 7.99 7.98 7.98 7.63 7.55 7.53 7.52 7.29 7.17 7.08 7.05 6.54 6.51 5.09 5.08 5.05 5.04 3.86 2.69 2.32 2.31 2.28 2.27 2.09 2.05 1.68
1.95 0.99 2.00 0.89 1.00 0.95 1.00 0.77 2.89 5.96 1.03 1.06 3.09
f1 (ppm)

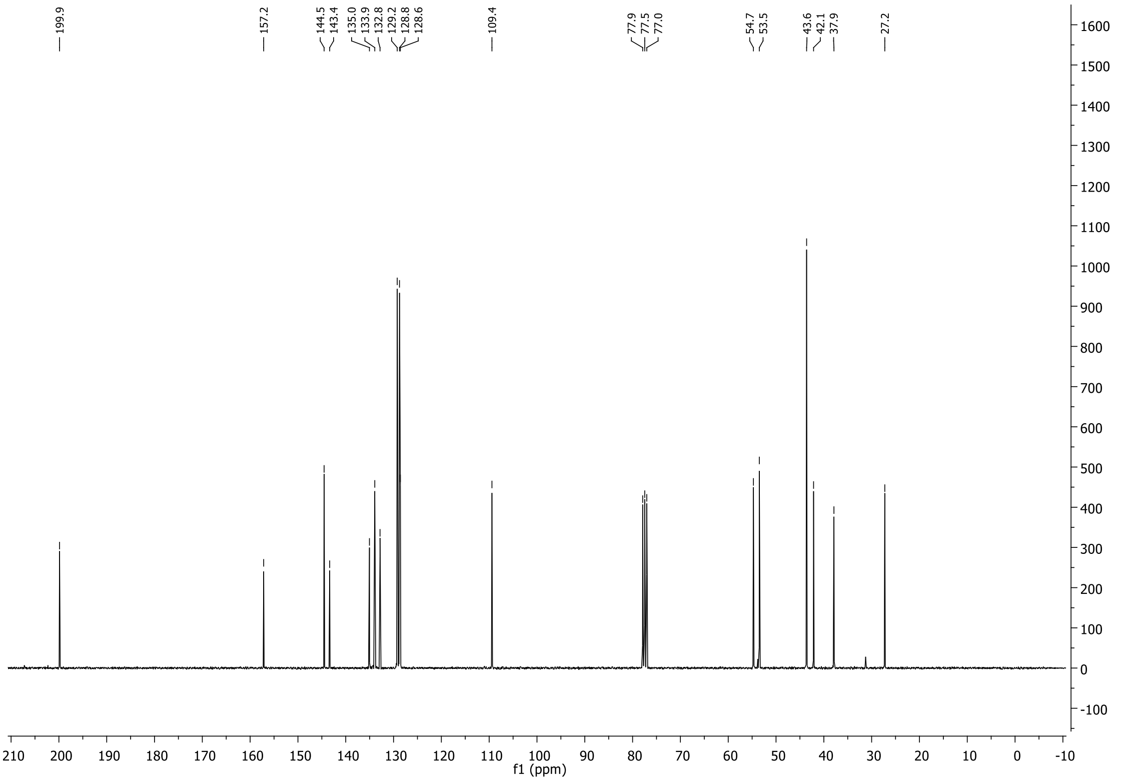
199.9 157.2 144.5 143.4 135.0 133.9 132.8 129.2 128.8 128.6 109.4 77.9 77.5 77.0 54.7 53.5 43.6 42.1 37.9 27.2
f1 (ppm)

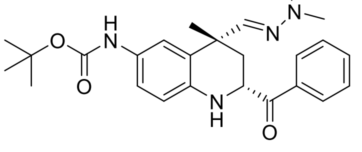
H
N
O
O
N
N
N
H
O

2f

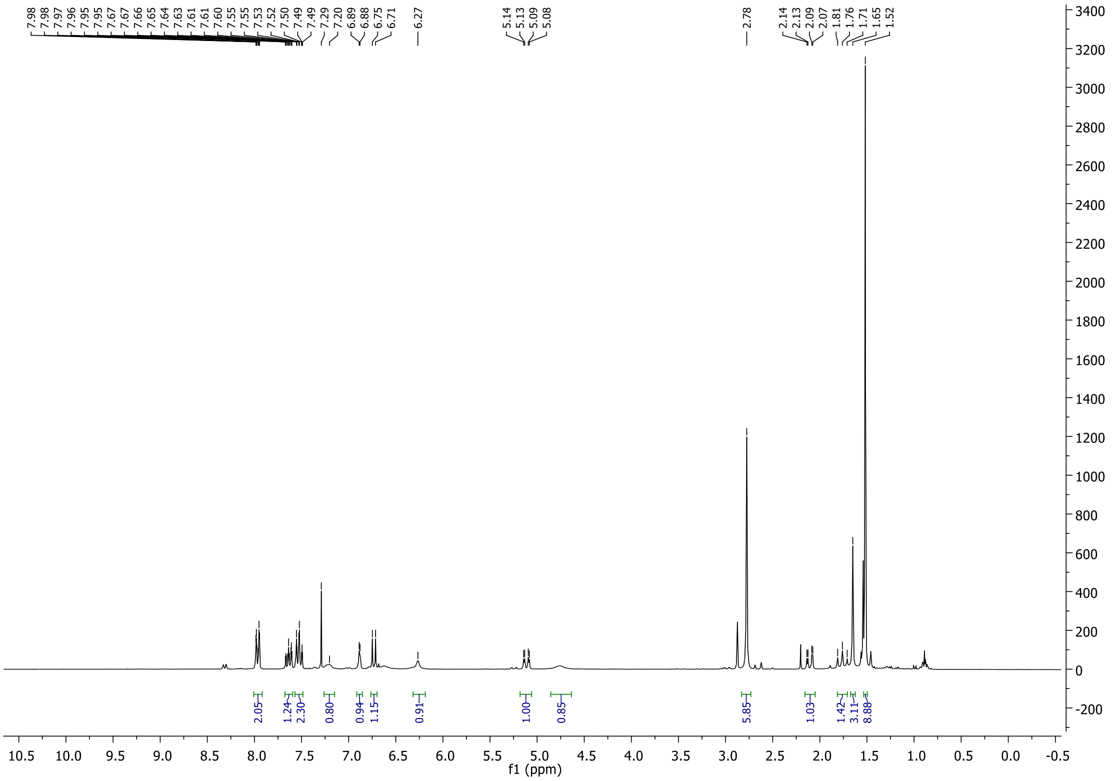
7.98 7.98 7.97 7.96 7.95 7.95 7.67 7.67 7.66 7.65 7.64 7.63 7.61 7.61 7.60 7.55 7.55 7.53 7.52 7.50 7.49 7.49 7.29 7.20 6.89 6.88 6.75 6.71 6.27
5.14 5.13 5.09 5.08
2.78
2.14 2.13 2.09 2.07 1.81 1.76 1.71 1.65 1.52
2.05 1.24 2.30 0.80 0.94 1.15 0.91 1.00 0.85 5.85 1.03 1.42 3.11 8.88
f1 (ppm)

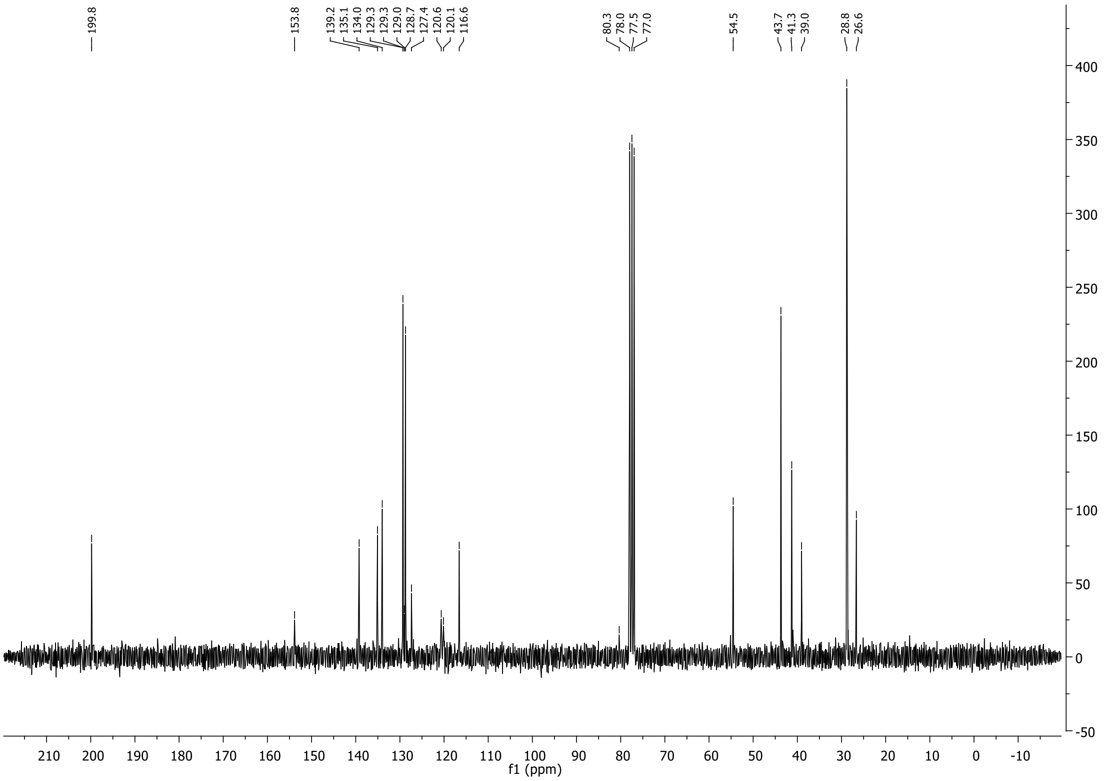
199.8
153.8
139.2 135.1 134.0 129.3 129.3 129.0 128.7 127.4 120.6 120.1 116.6
80.3 78.0 77.5 77.0
54.5
43.7 41.3 39.0
28.8 26.6
f1 (ppm)

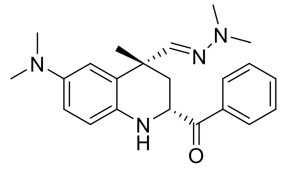
N
N
N
N
H
O

2g

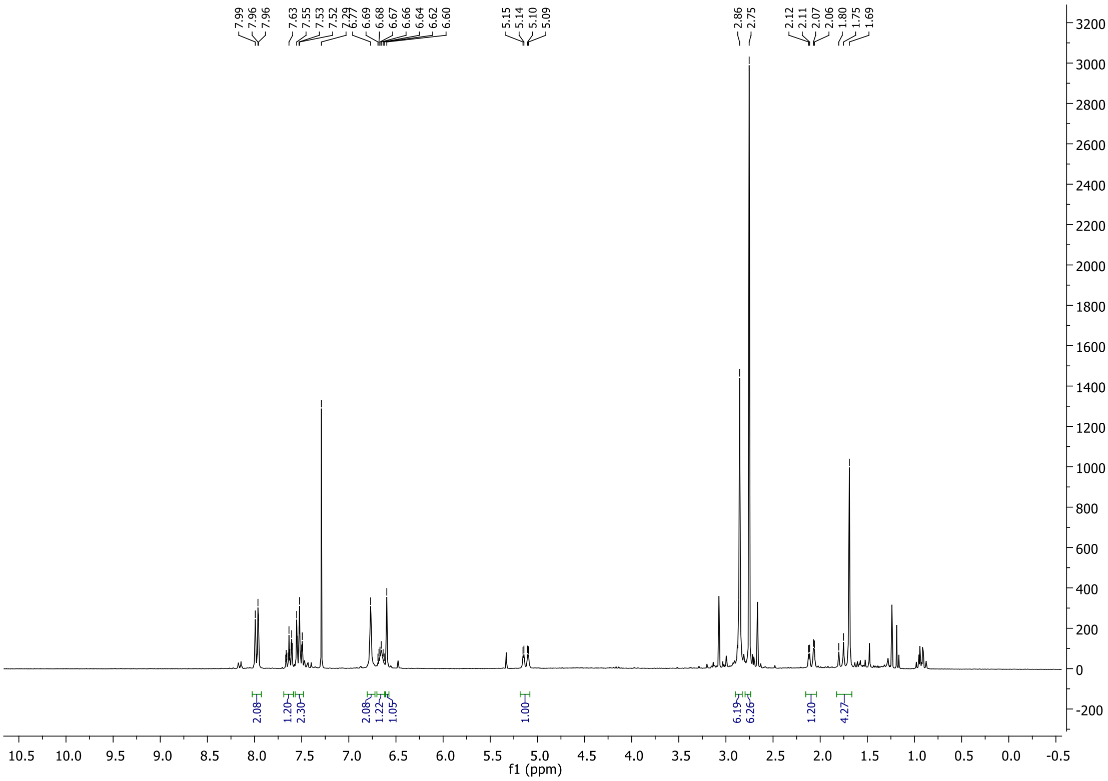
7.99
7.96
7.96
7.63
7.55
7.53
7.52
7.29
6.77
6.69
6.68
6.67
6.66
6.64
6.62
6.60
5.15
5.14
5.10
5.09
2.86
2.75
2.12
2.11
2.07
2.06
1.80
1.75
1.69
2.08
1.20
2.30
2.08
1.22
1.05
1.00
6.19
6.26
1.20
4.27
f1 (ppm)

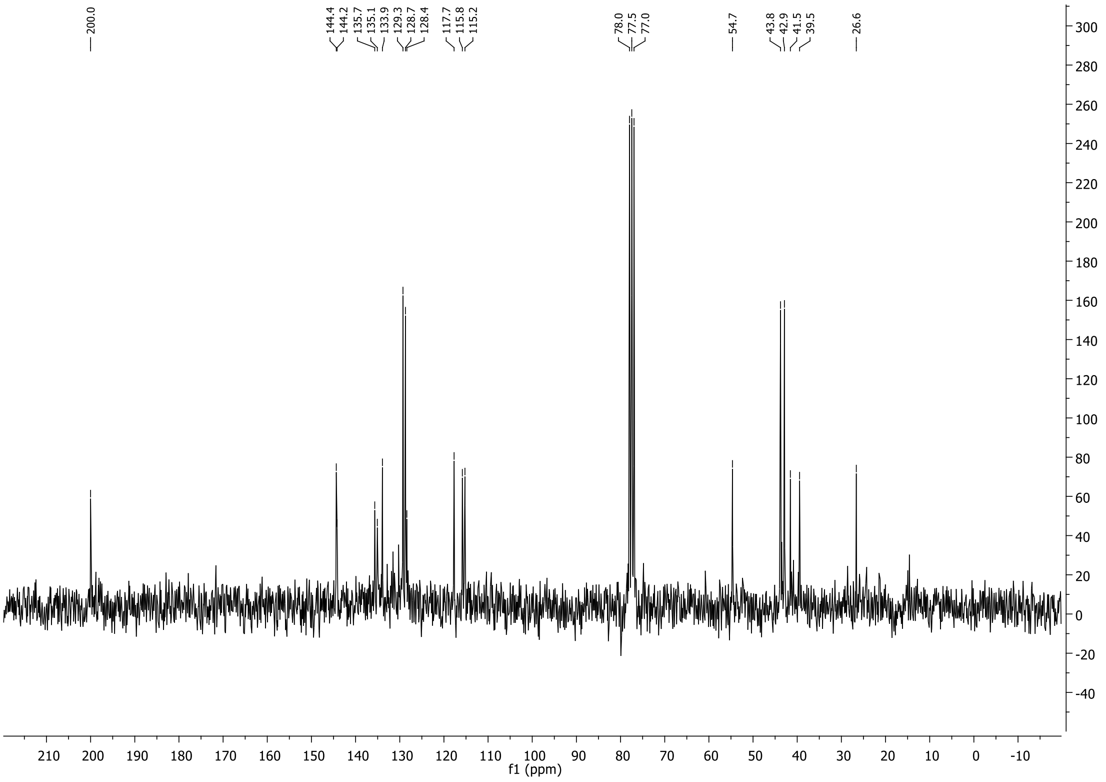
200.0
144.4
144.2
135.7
135.1
133.9
129.3
128.7
128.4
117.7
115.8
115.2
78.0
77.5
77.0
54.7
43.8
42.9
41.5
39.5
26.6
f1 (ppm)

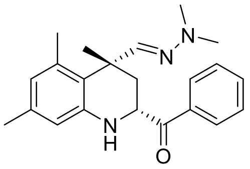
N
N
N
H
O

2h

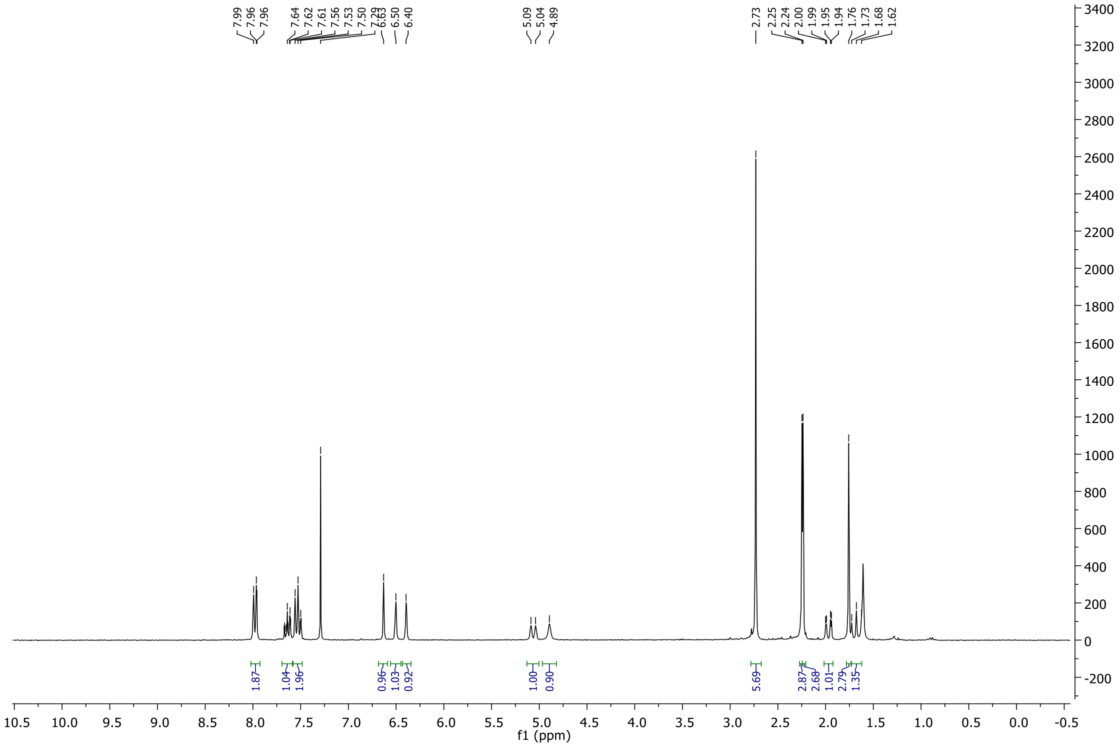
7.99
7.96
7.96
7.64
7.62
7.61
7.56
7.53
7.50
7.29
6.63
6.50
6.40
5.09
5.04
4.89
2.73
2.25
2.24
2.00
1.99
1.95
1.94
1.76
1.73
1.68
1.62
1.87
1.04
1.96
0.96
1.03
0.92
1.00
0.90
5.69
2.87
2.69
1.01
2.79
1.35
f1 (ppm)

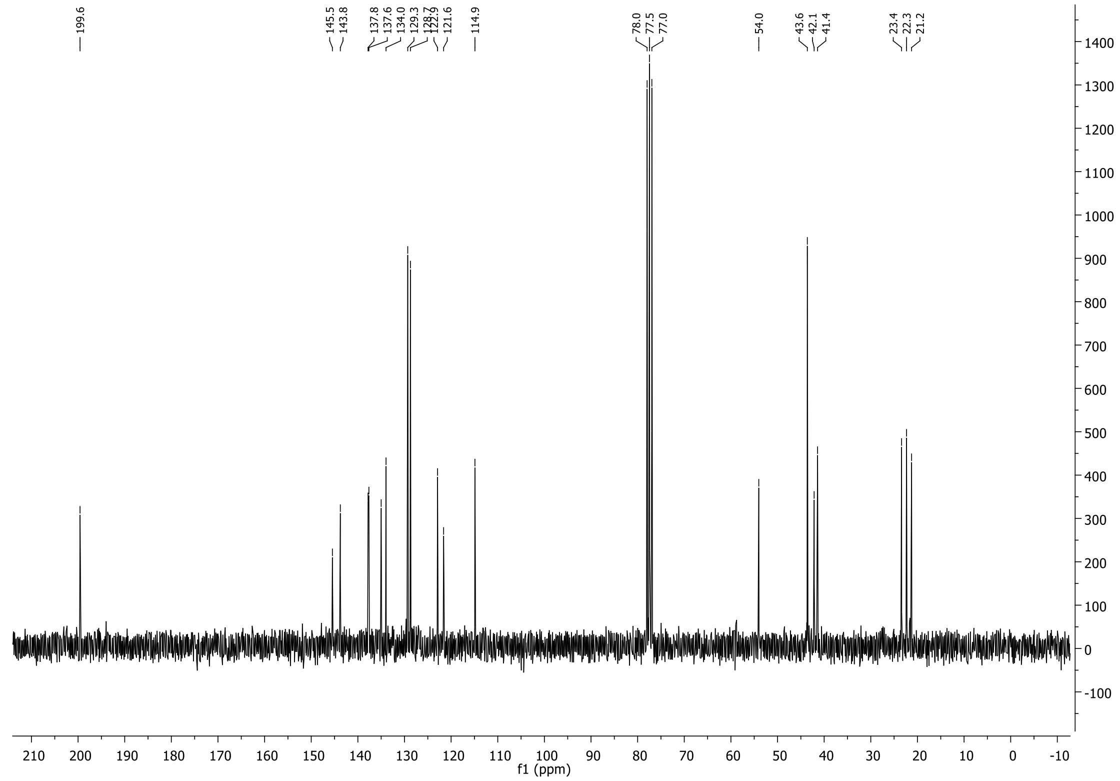
199.6
145.5
143.8
137.8
137.6
134.0
129.3
128.7
122.9
121.6
114.9
78.0
77.5
77.0
54.0
43.6
42.1
41.4
23.4
22.3
21.2
f1 (ppm)

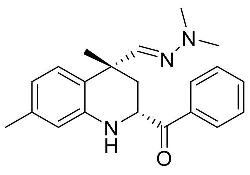
N
N
N
H
O

2i

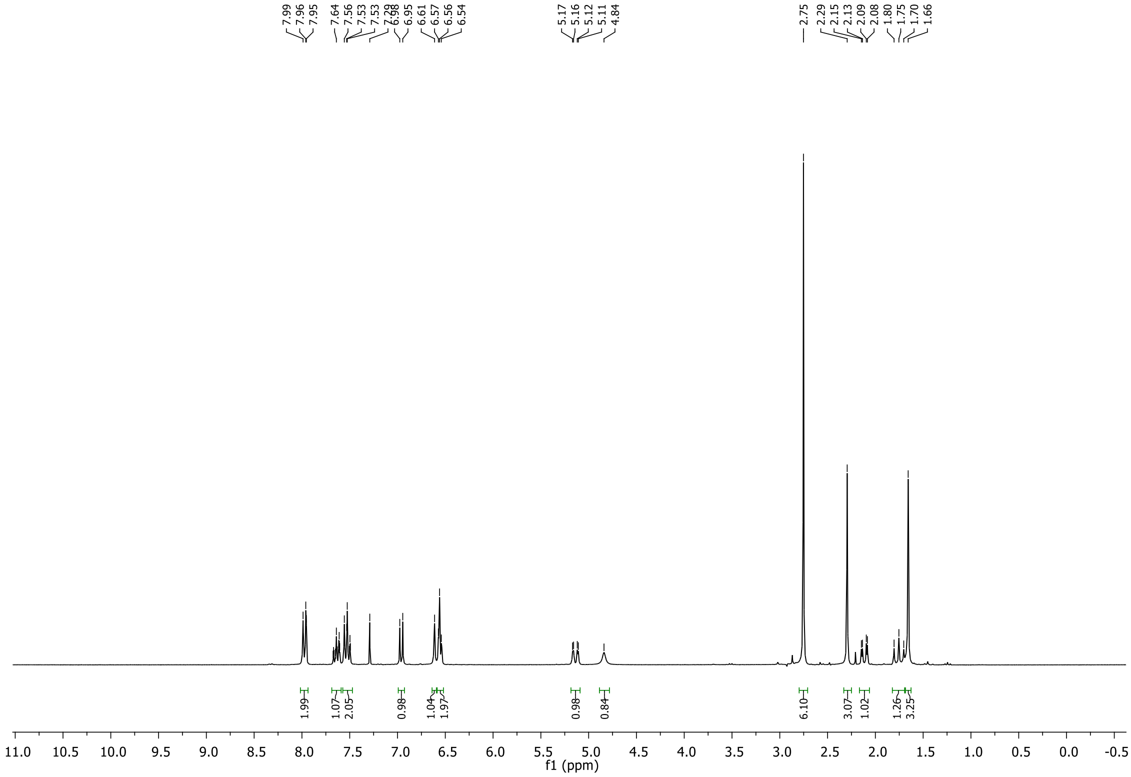
7.99 7.96 7.95 7.64 7.56 7.53 7.53 7.29 6.98 6.95 6.61 6.57 6.56 6.54
5.17 5.16 5.12 5.11 4.84
2.75 2.29 2.15 2.13 2.09 2.08 1.80 1.75 1.70 1.66
1.99 1.07 2.05 0.98 1.04 1.97 0.98 0.84 6.10 3.07 1.02 1.26 3.25
11.0 10.5 10.0 9.5 9.0 8.5 8.0 7.5 7.0 6.5 6.0 5.5 5.0 4.5 4.0 3.5 3.0 2.5 2.0 1.5 1.0 0.5 0.0 -0.5
f1 (ppm)

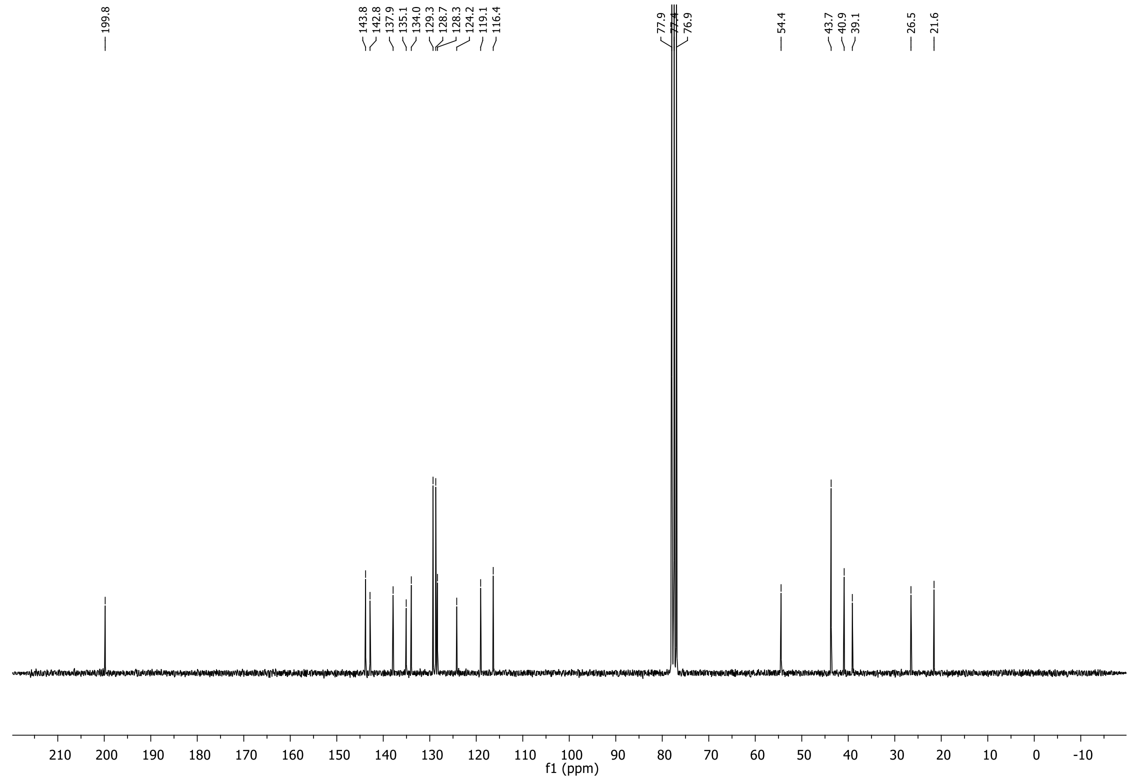
199.8
143.8 142.8 137.9 135.1 134.0 129.3 128.7 128.3 124.2 119.1 116.4
77.9 77.4 76.9
54.4
43.7 40.9 39.1
26.5 21.6
210 200 190 180 170 160 150 140 130 120 110 100 90 80 70 60 50 40 30 20 10 0 -10
f1 (ppm)

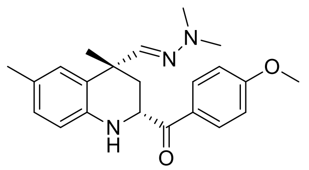

2k

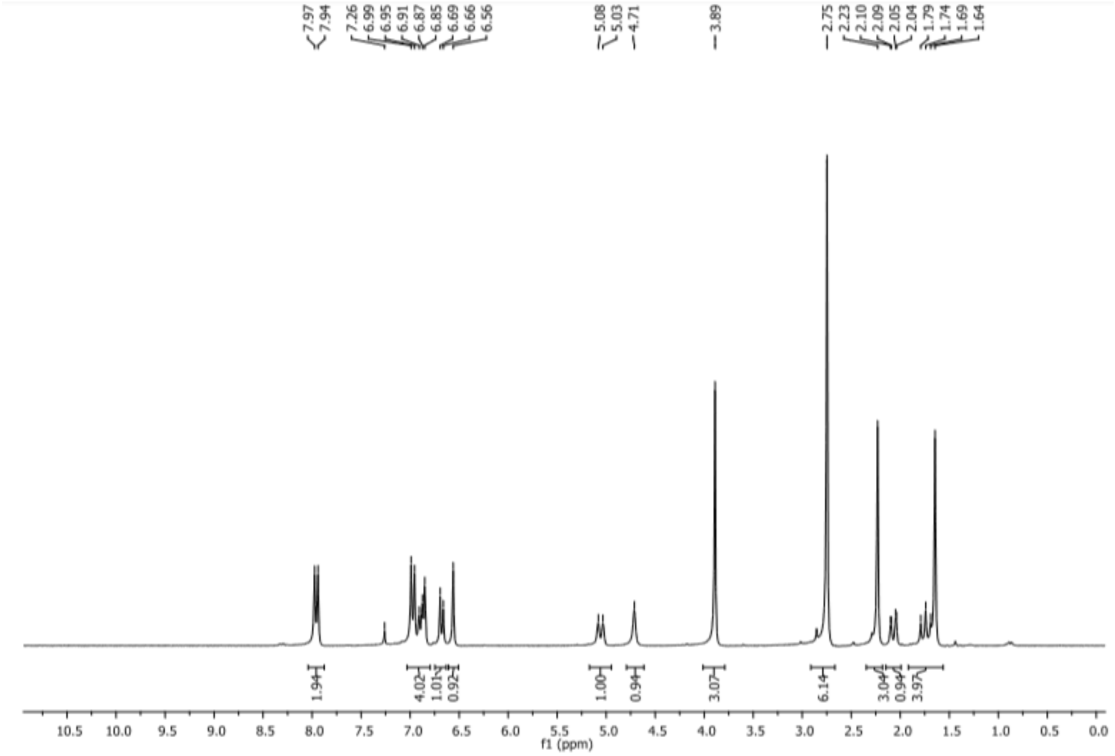

7.97
7.94
7.26
6.99
6.95
6.91
6.87
6.85
6.69
6.66
6.56
5.08
5.03
4.71
3.89
2.75
2.23
2.10
2.09
2.05
2.04
1.79
1.74
1.69
1.64
1.94
4.02
1.01
0.92
1.00
0.94
3.07
6.14
3.04
0.94
3.97
f1 (ppm)

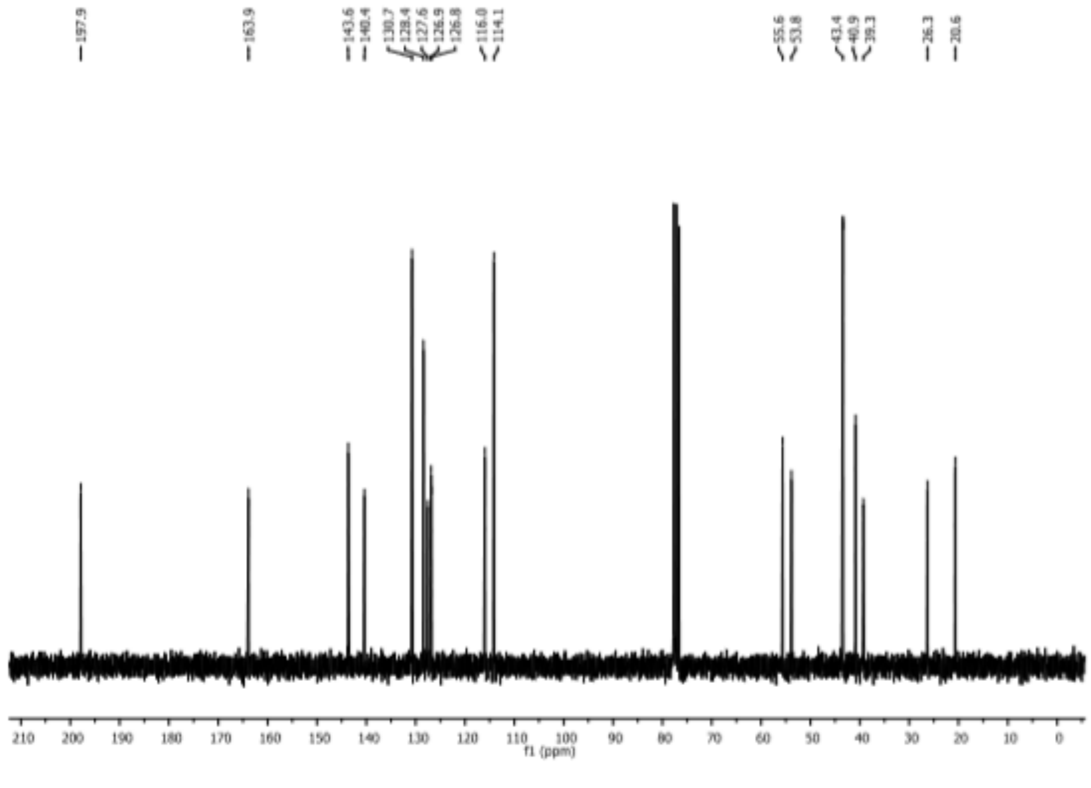

197.9
163.9
143.6
140.4
130.7
128.4
127.6
126.9
126.8
116.0
114.1
55.6
53.8
43.4
40.9
39.3
26.3
20.6
f1 (ppm)

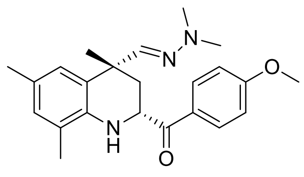

2l

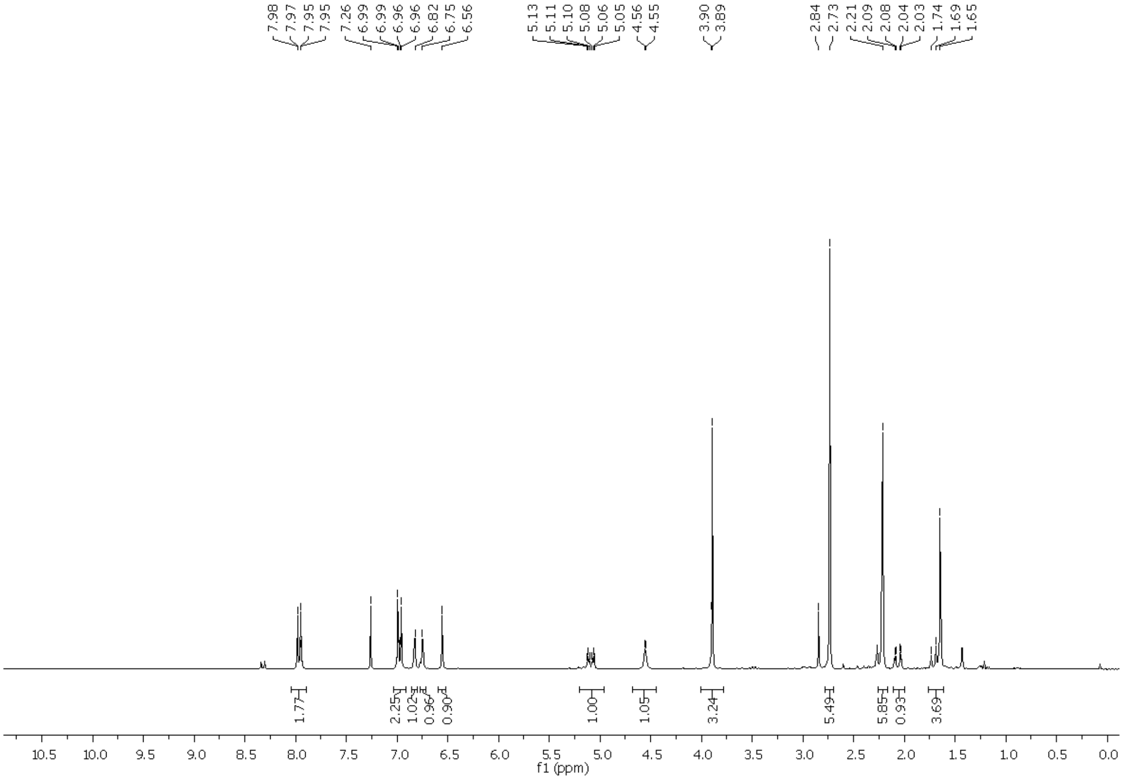
7.98 7.97 7.95 7.95 7.26 6.99 6.99 6.96 6.96 6.82 6.75 6.56 5.13 5.11 5.10 5.08 5.06 5.05 4.56 4.55 3.90 3.89 2.84 2.73 2.21 2.09 2.08 2.04 2.03 1.74 1.69 1.65
1.77 2.25 1.02 0.96 0.90 1.00 1.05 3.24 5.49 5.85 0.93 3.69
f1 (ppm)

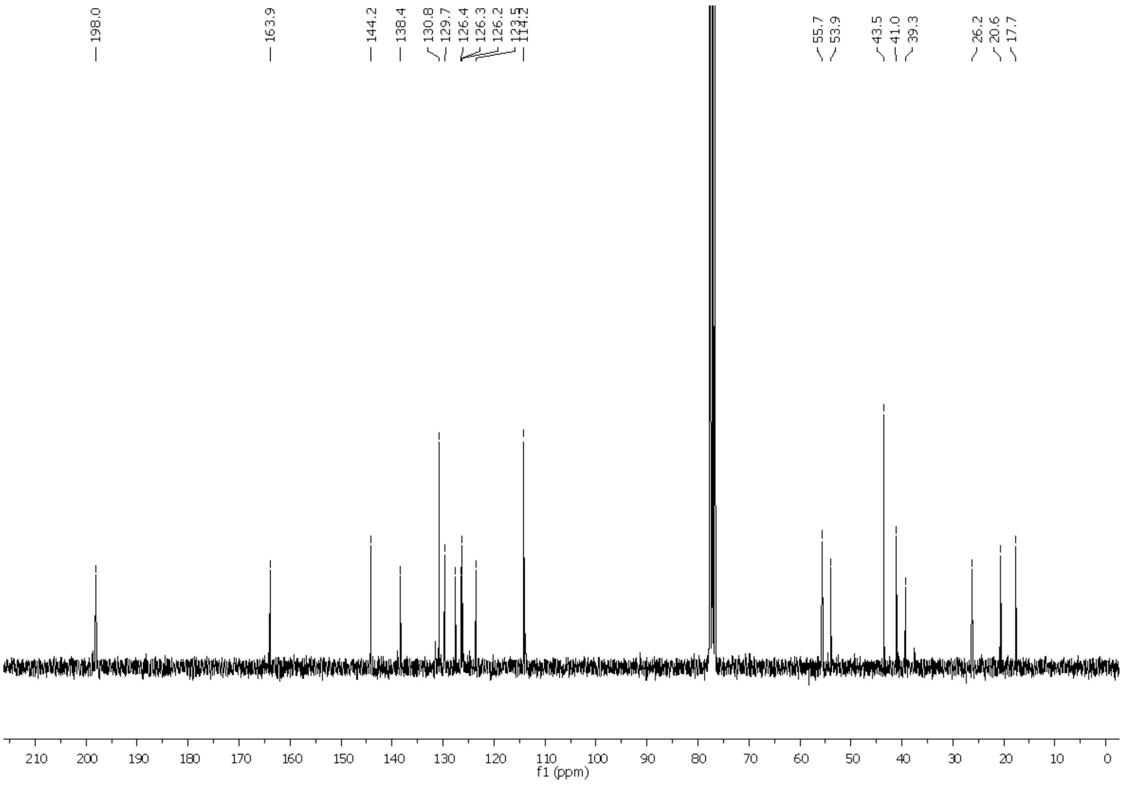
198.0 163.9 144.2 138.4 130.8 129.7 126.4 126.3 126.2 123.5 114.2 55.7 53.9 43.5 41.0 39.3 26.2 20.6 17.7
f1 (ppm)

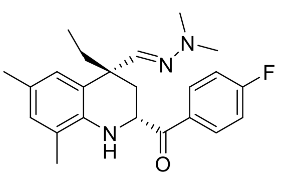
N
N
F
N
H
O

2n

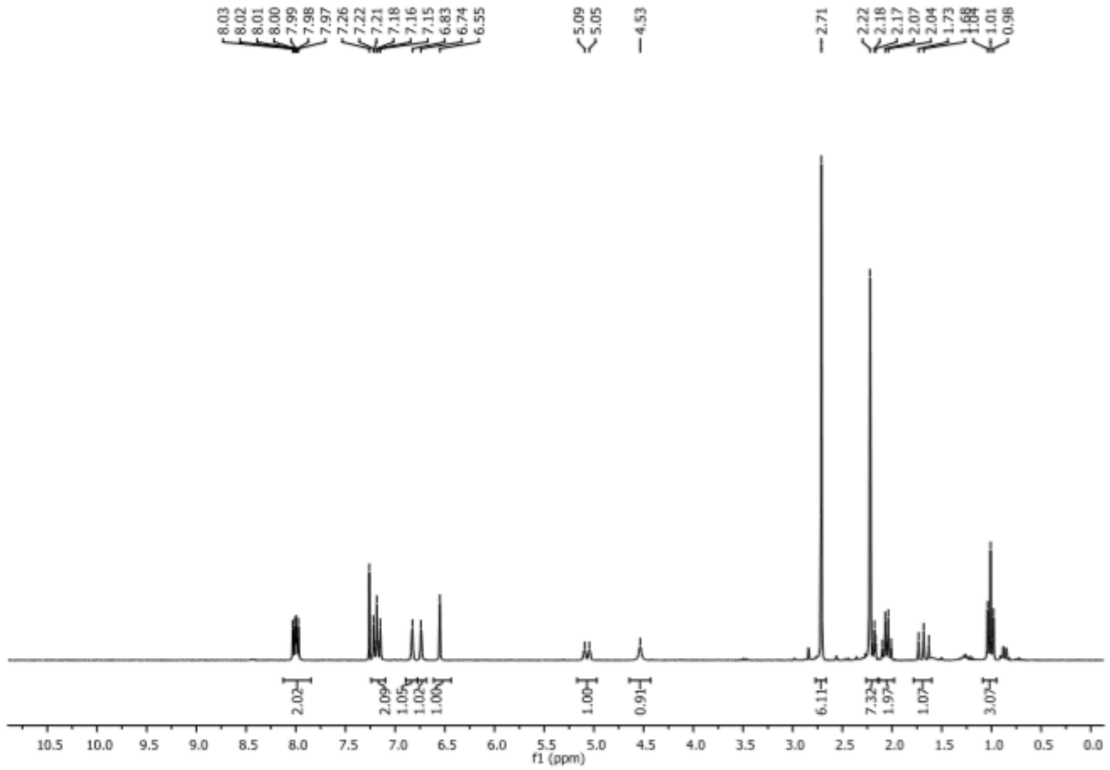
8.03
8.02
8.01
8.00
7.99
7.98
7.97
7.26
7.22
7.21
7.18
7.16
7.15
6.83
6.74
6.55
5.09
5.05
4.53
2.71
2.22
2.18
2.17
2.07
2.04
1.73
1.68
1.01
0.98
2.02
2.09
1.05
1.02
1.00
1.00
0.91
6.11
7.32
1.97
1.07
3.07
f1 (ppm)

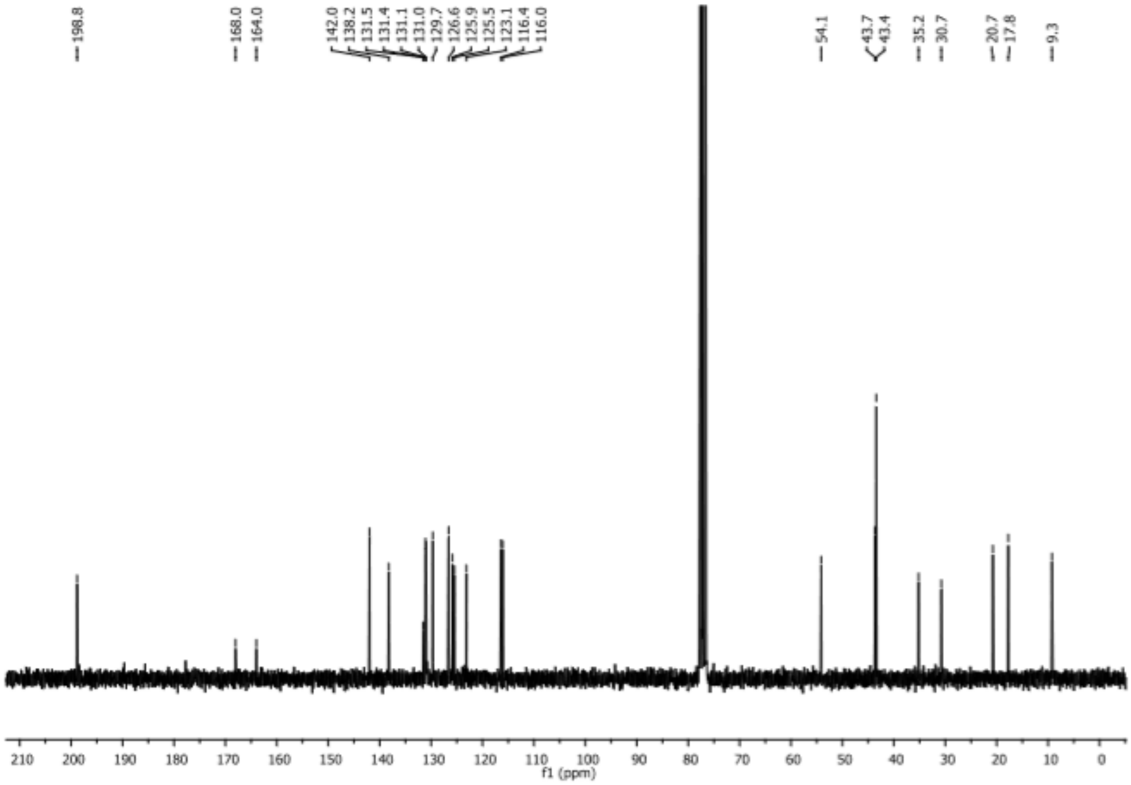
198.8
168.0
164.0
142.0
138.2
131.5
131.4
131.1
131.0
129.7
126.6
125.9
125.5
123.1
116.4
116.0
54.1
43.7
43.4
35.2
30.7
20.7
17.8
9.3
f1 (ppm)

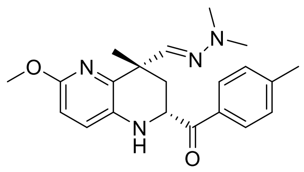
O
N
N
N
N
H
O

2o

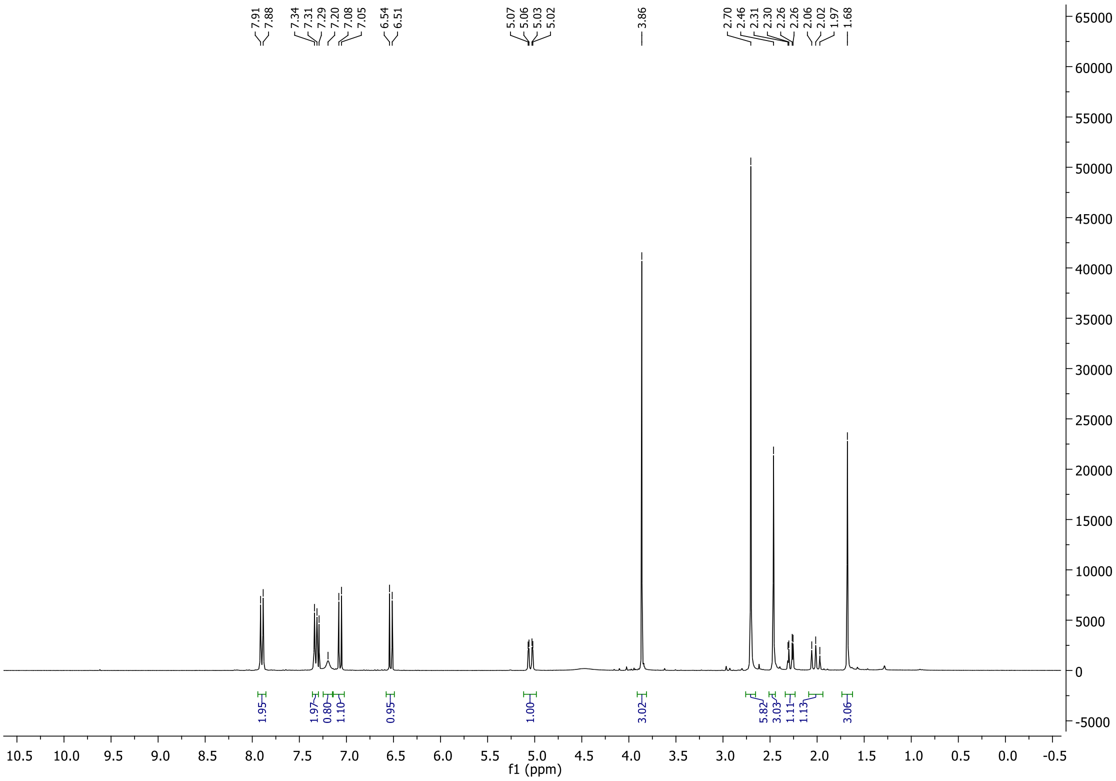
7.91
7.88
7.34
7.31
7.29
7.20
7.08
7.05
6.54
6.51
5.07
5.06
5.03
5.02
3.86
2.70
2.46
2.31
2.30
2.26
2.26
2.06
2.02
1.97
1.68
1.95
1.97
0.80
1.10
0.95
1.00
3.02
5.82
3.03
1.11
1.13
3.06
f1 (ppm)

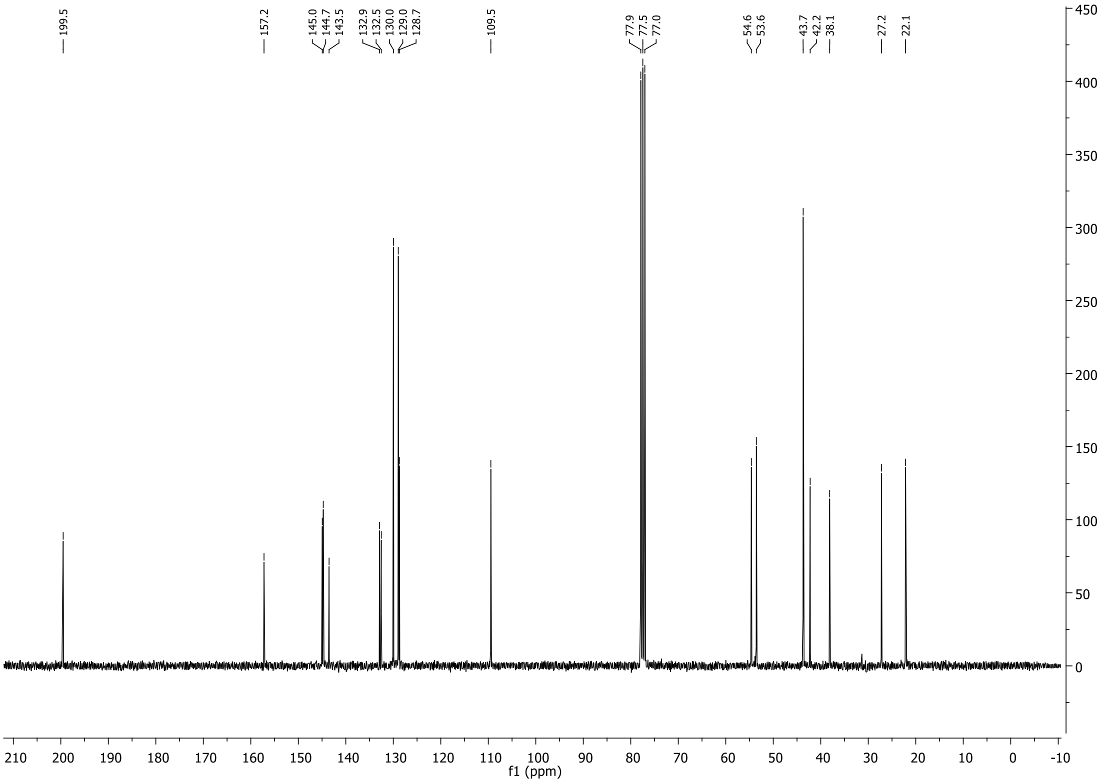
199.5
157.2
145.0
144.7
143.5
132.9
132.5
130.0
129.0
128.7
109.5
77.9
77.5
77.0
54.6
53.6
43.7
42.2
38.1
27.2
22.1
f1 (ppm)

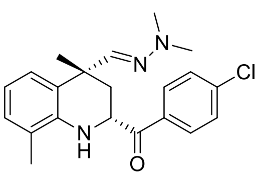
N
N
Cl
N
H
O

2p

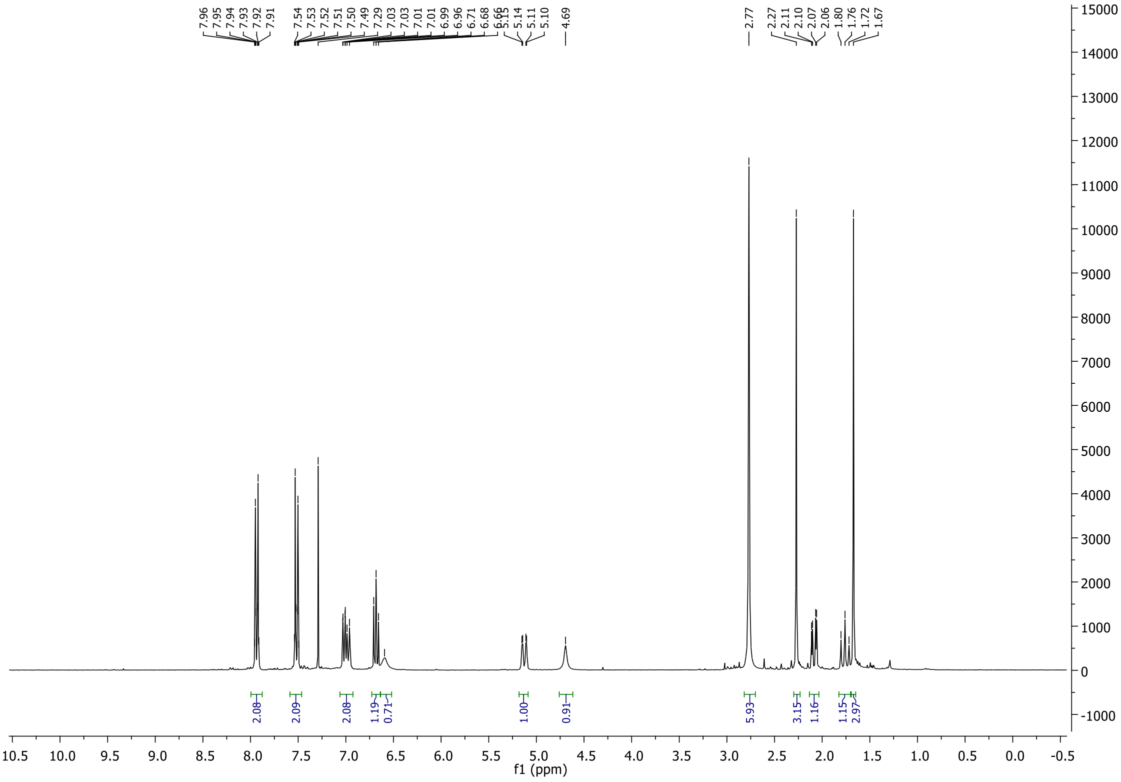
7.96 7.95 7.94 7.93 7.92 7.91 7.54 7.53 7.52 7.51 7.50 7.49 7.29 7.03 7.03 7.01 7.01 6.99 6.96 6.71 6.68 6.66 5.15 5.14 5.11 5.10 4.69
2.77 2.27 2.11 2.10 2.07 2.06 1.80 1.76 1.72 1.67
2.08 2.09 2.08 1.19 0.71 1.00 0.91 5.93 3.15 1.16 1.15 2.97
f1 (ppm)

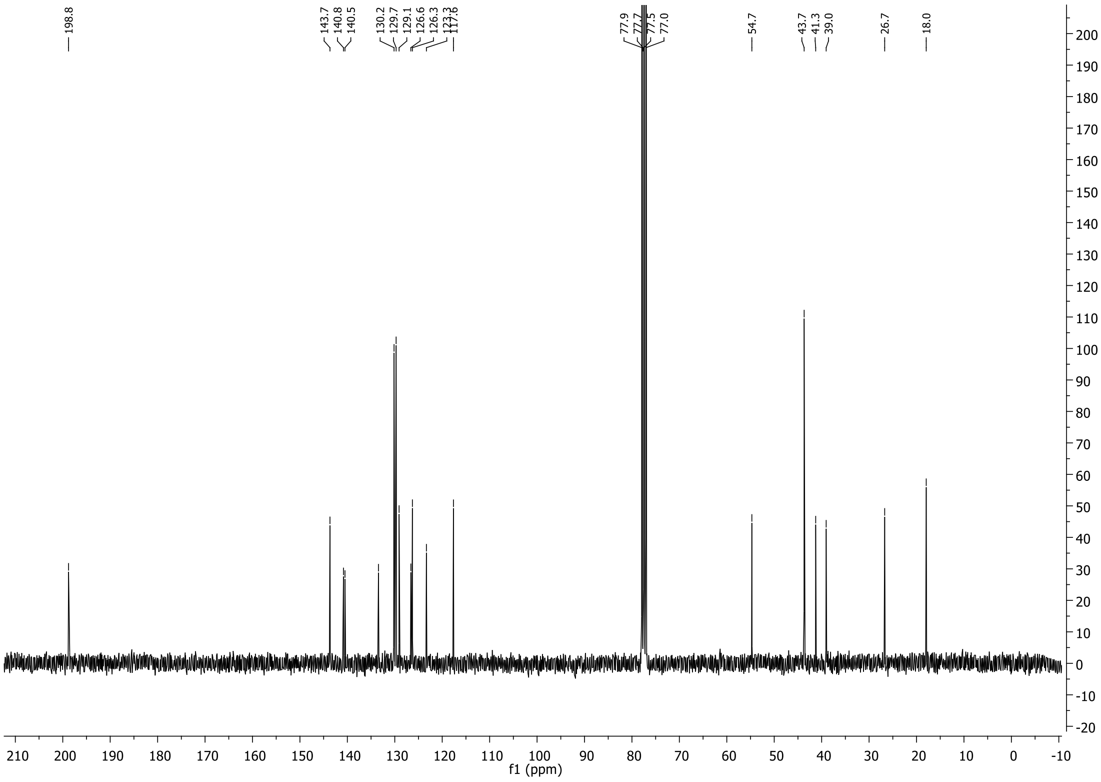
198.8
143.7 140.8 140.5 130.2 129.7 129.1 126.6 126.3 123.3 117.6
77.9 77.7 77.5 77.0
54.7
43.7 41.3 39.0
26.7
18.0
f1 (ppm)

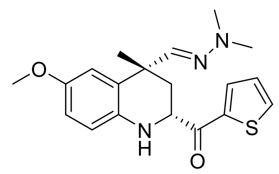

2s

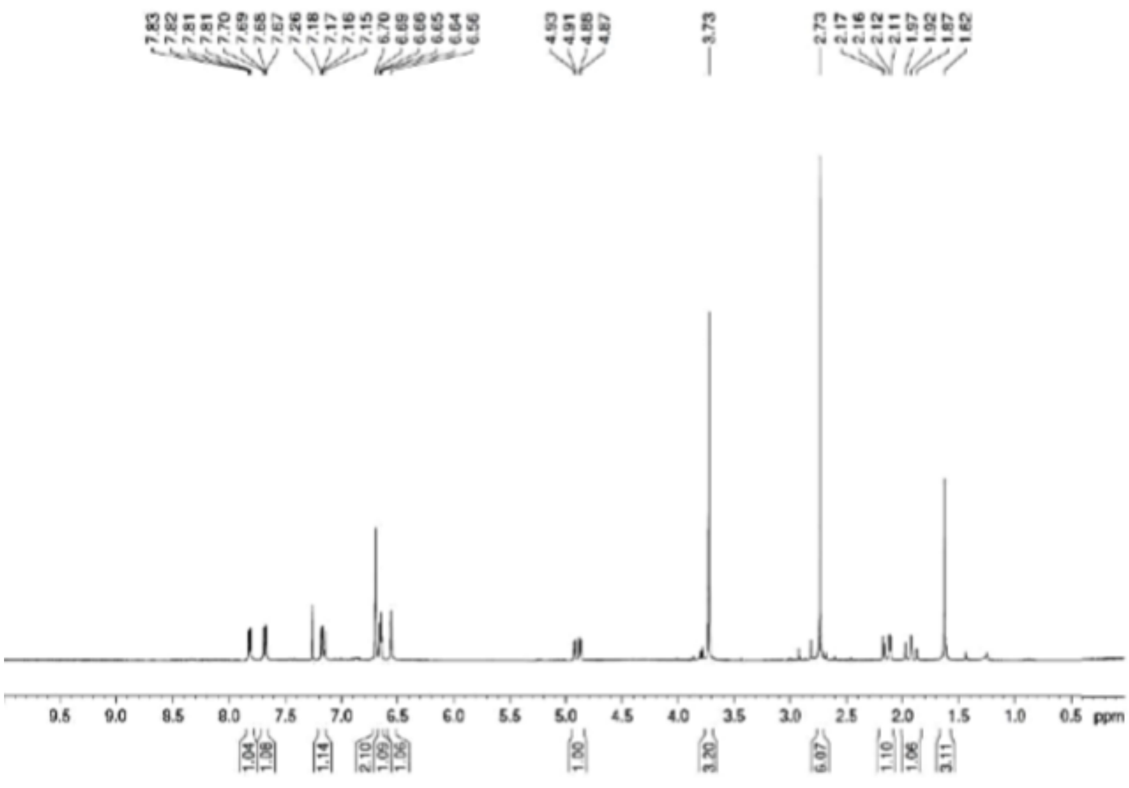
7.83 7.82 7.81 7.81 7.70 7.69 7.68 7.67 7.26 7.18 7.17 7.16 7.15 6.70 6.69 6.66 6.65 6.64 6.56
4.93 4.91 4.88 4.87
3.73
2.73 2.17 2.16 2.12 2.11 1.97 1.92 1.87 1.62
9.5 9.0 8.5 8.0 7.5 7.0 6.5 6.0 5.5 5.0 4.5 4.0 3.5 3.0 2.5 2.0 1.5 1.0 0.5 ppm
1.04 1.08 1.14 2.10 1.09 1.06 1.00 3.20 6.07 1.10 1.08 3.11

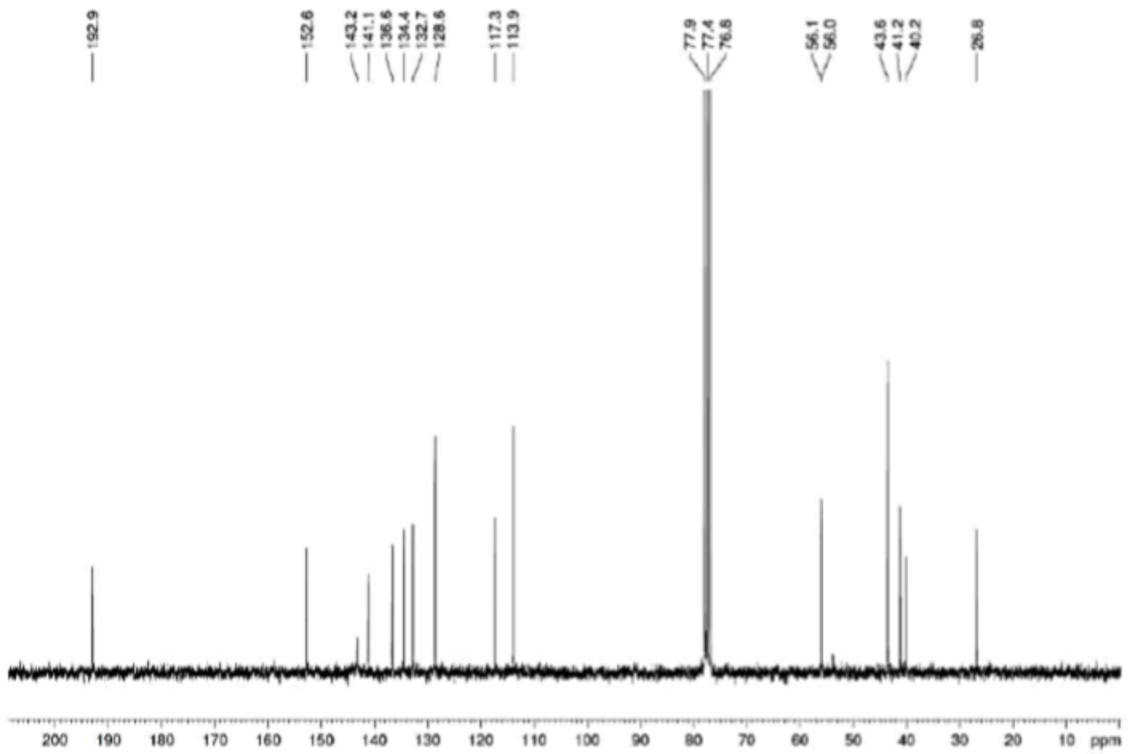
192.9
152.6
143.2 141.1 136.6 134.4 132.7 128.6
117.3 113.9
77.9 77.4 76.8
56.1 56.0
43.6 41.2 40.2
26.8
200 190 180 170 160 150 140 130 120 110 100 90 80 70 60 50 40 30 20 10 ppm
